# Beyond the G protein α subunit: investigating the functional impact of other components of the Gαi_3_ heterotrimers

**DOI:** 10.1186/s12964-023-01307-w

**Published:** 2023-10-10

**Authors:** Beata Rysiewicz, Ewa Błasiak, Paweł Mystek, Marta Dziedzicka-Wasylewska, Agnieszka Polit

**Affiliations:** Department of Physical Biochemistry, Faculty of Biochemistry Biophysics and Biotechnology, Jagiellonian University, Gronostajowa 7, 30-387 Kraków, Poland

**Keywords:** Heterotrimeric G proteins, Dopamine D_2_ receptor, GPCR, Gαi_3_ subunit, Gβγ dimer, Subcellular localization, Live-cell imaging, FRET, Intracellular cAMP levels, Docking simulations

## Abstract

**Background:**

Specific interactions between G protein-coupled receptors (GPCRs) and G proteins play a key role in mediating signaling events. While there is little doubt regarding receptor preference for Gα subunits, the preferences for specific Gβ and Gγ subunits and the effects of different Gβγ dimer compositions on GPCR signaling are poorly understood. In this study, we aimed to investigate the subcellular localization and functional response of Gαi_3_-based heterotrimers with different combinations of Gβ and Gγ subunits.

**Methods:**

Live-cell imaging microscopy and colocalization analysis were used to investigate the subcellular localization of Gαi_3_ in combination with Gβ_1_ or Gβ_2_ heterotrimers, along with representative Gγ subunits. Furthermore, fluorescence lifetime imaging microscopy (FLIM-FRET) was used to investigate the nanoscale distribution of Gαi_3_-based heterotrimers in the plasma membrane, specifically with the dopamine D_2_ receptor (D_2_R). In addition, the functional response of the system was assessed by monitoring intracellular cAMP levels and conducting bioinformatics analysis to further characterize the heterotrimer complexes.

**Results:**

Our results show that Gαi_3_ heterotrimers mainly localize to the plasma membrane, although the degree of colocalization is influenced by the accompanying Gβ and Gγ subunits. Heterotrimers containing Gβ_2_ showed slightly lower membrane localization compared to those containing Gβ_1_, but certain combinations, such as Gαi_3_β_2_γ_8_ and Gαi_3_β_2_γ_10_, deviated from this trend. Examination of the spatial arrangement of Gαi_3_ in relation to D_2_R and of changes in intracellular cAMP level showed that the strongest functional response is observed for those trimers for which the distance between the receptor and the Gα subunit is smallest, i.e. complexes containing Gβ_1_ and Gγ_8_ or Gγ_10_ subunit. Deprivation of Gαi_3_ lipid modifications resulted in a significant decrease in the amount of protein present in the cell membrane, but did not always affect intracellular cAMP levels.

**Conclusion:**

Our studies show that the composition of G protein heterotrimers has a significant impact on the strength and specificity of GPCR-mediated signaling. Different heterotrimers may exhibit different conformations, which further affects the interactions of heterotrimers and GPCRs, as well as their interactions with membrane lipids. This study contributes to the understanding of the complex signaling mechanisms underlying GPCR-G-protein interactions and highlights the importance of the diversity of Gβ and Gγ subunits in G-protein signaling pathways.

Video Abstract

**Supplementary Information:**

The online version contains supplementary material available at 10.1186/s12964-023-01307-w.

## Background

G protein-coupled receptors (GPCRs), which are encoded by more than 800 genes in the human genome, constitute a huge group of eukaryotic transmembrane proteins. Their primary function is to transmit extracellular signals into the cell, allowing cells to communicate with each other and sense the extracellular environment. Unlike other types of receptors, GPCRs rely on interactions with G proteins, which further transmit signals to membrane-bound effectors. G proteins play a key role in providing the high flexibility, sensitivity, and specificity observed in GPCR signaling. Upon activation by various ligands, GPCRs can regulate a wide range of signaling pathways by engaging small G-proteins and heterotrimeric guanine nucleotide-binding proteins, referred to as G-proteins hereafter. The heterotrimeric G-protein complex consists of one of 16 Gα subunits, one of 5 Gβ subunits, and one of 12 Gγ subunits [1]. Considering that all possible combinations of these three subunits can likely form within cells, the number of potential heterotrimeric combinations is extensive. The diverse signaling properties of GPCRs account for the multitude of stimuli and cellular responses they are involved in. This diversity is achieved by the diversity in the varied composition of G protein heterotrimers, thereby contributing to the complexity of the human organism. However, it also gives rise to intriguing questions, such as how the appropriate receptor interacts with the specific G-protein trimer in the precise cellular context.

G protein heterotrimers function as regulatory GTP hydrolases. Based on the sequence similarities of the Gα subunit, they can be categorized into four groups: Gαs, Gαi/o, Gαq/11, and Gα12/13. Each family member within these groups acts on distinct signaling pathways, thereby modulating various physiological processes in response to extracellular stimuli. The Gαs family subunits stimulate adenylate cyclases (ACs), while different Gαi/o subunits exert an inhibitory effect on specific ACs. The Gαq/11 subfamily regulates phospholipase C activity, and Gα12/13 can interact with Rho nucleotide exchange factors [2]. In the GDP-bound conformation, the βγ dimer prevents the release of the nucleotide, thereby stabilizing the inactive form of the Gαβγ heterotrimer. When an activated GPCR receptor binds to it, a rapid release of GDP and its replacement by GTP is induced. This results in the dissociation of both the GPCR–Gαβγ complex and the rearrangement of the G-protein complex into the free Gα subunit and the Gβγ complex, which can independently activate different signal transduction pathways, leading to specific physiological effects. Once GTP is hydrolyzed to GDP by the Gα subunit, the now inactive Gα subunit can reassociate with the Gβγ dimer.

The diversity of signals sent into the cell following GPCR activation arises from the GPCRs’ capacity to activate multiple G proteins [3]. Most GPCRs interact with specific Gα subunits, which in turn determine distinct patterns of engagement with effector molecules [2]. However, GPCRs can activate any G protein, albeit with varying efficiencies [4]. The picture gets even more complicated when we consider that Gγ subunits regulate the spatial–temporal organization of G proteins. Since all Gβγ complexes can dissociate from the cell membrane, the membrane dissociation of Gβγ complexes serves as an additional mechanism to control their availability within the submembrane space. Consequently, cells expressing different levels of Gγ subunits exhibit distinct characteristics in their response to GPCR stimulation.

The existence of so many combinations of Gβγ complexes and their function in cells remains unexplained, as for many years this complex has been shadowed by research on the Gα subunit. But this is the Gβγ complex, which—upon heterotrimer activation—changes its localization and is responsible for effects in areas distant from the membrane. As it turns out, different combinations of Gβ and Gγ subunits can combine to form complexes with different affinities, the ability to move to distinct cellular compartments, and generate different outcomes [4]. So, it seems likely that the diversity in the composition of Gβγ complexes is responsible for the plasticity of GPCRs to generate intracellular responses. Transfer of the Gβγ complex from the plasma membrane determines signaling to intracellular organelles and can proceed in two ways. Rapidly dissociating farnesylated Gγ promptly diffuses into the endoplasmic reticulum (ER), Golgi apparatus, mitochondrion, and early endosomes. Geranylated Gγ subunits can only reach the ER and mitochondrion by slow diffusion, or with active transport to the early endosomes and Golgi [4]. The rate of translocation of human Gγ-subunits of G proteins from the plasma membrane to inner membranes has, incidentally, been used as the basis for one of two classifications of these proteins, according to which it distinguishes fast-moving subunits (about 10 s), subunits moving at an intermediate rate (about 60 s) and subunits moving slowly (more than 2 min) [5]. The second classification method, which we follow in the present study, assigns these proteins to five classes based on sequence homology, and so Class I includes Gγ_1_, Gγ_9,_ and Gγ_11_, Class II: Gγ_2_, Gγ_8_, Gγ_3_, and Gγ_4_, Class III: Gγ_7_ and Gγ_12_, Class IV: Gγ_5_ and Gγ_10_ and Class V: Gγ_13_ [6].

The precise determinants of the interaction between receptors and G proteins are not yet fully elucidated. However, it is well-established that the second and third intracellular loops of the receptor, as well as the third, fifth, and sixth transmembrane helices, exhibit significant structural diversity among GPCRs. These regions are responsible for the selective binding of the Gα subunits [7]. On the G protein side, the C-terminal and N-terminal helices, along with two loops of the Gα subunit, are involved in the interaction with receptors. The interaction between the receptor immobilized in the membrane and the Gαi subunit is facilitated by lipid modifications that all G protein α-subunits undergo. Depending on the protein class, myristic and/or palmitic acid residues are attached. In the case of Gαi subunits, N-myristoylation, and S-palmitoylation take place. N-myristoylation is an irreversible process that anchors the subunit to the cell membrane. S-palmitoylation, on the other hand, is reversible and enables the regulation of protein accessibility to the receptor at different stages of protein transfer [8]. Additionally, all Gα subunits undergo co-translational irreversible N-terminal acylation. Furthermore, all Gγ subunits are modified at their C-terminus with a farnesyl or geranylgeranyl moiety [4]. Although Gα and Gγ subunits do not directly interact, their modified ends remain in close proximity and are responsible for the association of the complex with the membrane.

The Gβγ complex also binds to GPCRs, and this interaction stabilizes the GPCR-Gαβγ interface, presumably by placing Gα in a conformation suitable for receptor binding [9]. Experimental evidence has demonstrated the direct interaction of GPCRs with the C-terminus of the Gβ subunit and the farnesylated C-terminus of Gγ [10, 11]. Following activation, Gβγ dimers translocate to intracellular organelles to propagate the signaling from the cell membrane to these locations. The destination, kinetics, and efficiency of this translocation process are influenced by the specific Gγ subunit involved [4, 12, 13].

Recent evidence suggests that in addition to structural compatibility, the interaction between these proteins is influenced by various factors. These factors include the specific ligand used to activate the receptor, receptor oligomerization, and the lipid composition of the cell membrane [5, 14, 15]. This intricate signaling mechanism allows for diverse messages to be conveyed from the same receptor, leading to distinct signaling pathways and cellular responses. Different cell types exhibit variations in lipid composition and the expression of enzymes involved in lipid modification. Furthermore, within a single cell, different regions of the cell membrane can show heterogeneity in protein and lipid composition, including cholesterol and phospholipids, which can impact GPCR signaling [16, 17].

In the research described in the present studies, we focused on examining the impact of individual components of the heterotrimeric complex on the signaling of dopamine D_2_ receptors (D_2_R), which belong to the class A GPCRs. The stimulation of D_2_R leads to a reduction in cAMP levels through its interaction with Gαi/o class proteins. D_2_R is expressed in various brain regions, and it has been observed that Gαi subunits are also present in these regions without indicating regional specificity. According to the Human Protein Atlas, Gβ and Gγ subunits are also found in all brain structures without showing regional specificity, although their expression levels vary. Among the Gβ subunits, Gβ_1_ and Gβ_2_ have been reported to have the highest expression levels, while Gγ_3_, Gγ_8_, and Gγ_7_ are among the Gγ subunits with the highest expression levels (www.proteinatlas.org; [18]).

Using confocal microscopy and functional studies in living cells, as well as bioinformatics tools, we showed the composition of the heterocomplex, particularly the Gβ and Gγ subunits as well as lipid modifications. Our findings revealed that these factors are crucial in determining the localization of the heterocomplex within the cell membrane, the spatial orientation of the Gαi_3_ subunit relative to the dopamine D_2_R, and the cellular response upon receptor stimulation.

## Materials and methods

### Plasmid vectors and protein constructs

Plasmid pcDNA3.1+ vectors, namely GNB01, GNG01, GNG02, and GNG11, were obtained from the UMR cDNA Resource Center. Plasmids GNB02, GNG08, GNG09, and GNG13 were generously provided by Narasimhan Gautam (Addgene plasmids #42,182, #36,106, #64,203, #36,110). Plasmids GNG07, GNG10, and GNG12 were kindly provided by Catherine Berlot (Addgene plasmids #54,473, #55,192, #55,194) [5, 19]. Additionally, previously prepared and described plasmids were used in the studies described in this paper: pmCherry-N1DRD2, pcDNA3.1+ GNAI3Citrine, and pcDNA3.1+ GNASCitrine [20–22]. Plasmids containing GNAI3 and GNAS were modified to eliminate lipid moiety attachment through point mutations (G2A or C3A) using the QuikChange approach [23]. Sigma-Aldrich prepared starters (Poznań, Poland). The modified plasmids were confirmed by sequencing performed at Genomed (Warsaw, Poland).

### Cell culture and transfection

HEK293 cells (ATCC, Manassas, VA, USA) were cultured following previously described methods. For microscopy experiments, the cells were cultured on sterile glass coverslips in 30 mm plates. For cAMP and RT-qPCR assays, the cells were cultured in six-well plates, for cAMP assays additionally coated with 0.5% gelatin [21]. Transient transfection of the cells was performed using the TransITX2® Dynamic Delivery System (Mirus Bio, Madison, WI, USA) according to the manufacturer’s instructions. The transfection involved the use of pcDNA3.1+ GNAI3Citrine or GNASCitrine plasmids, as well as plasmids encoding Gβ and Gγ proteins, and pmCherry-N1DRD2. The DNA amounts for Gα, Gβ, and Gγ were equimolar in all experiments. The DNA ratio of Gαβγ with the D_2_R (used for cAMP level measurements) was 1–1.25. The total amount of DNA used for cell imaging experiments was 0.1–0.3 μg per dish, while for cAMP level determination and real-time PCR analysis, it was 1.7 μg per well.

Cell membrane staining was performed using the Cell Plasma Membrane Staining Kit Deep Red Fluorescence Cytopainter (ab219942; Abcam, Cambridge, GB) immediately before live-cell imaging according to the manufacturer’s instructions. After staining, the cell culture was washed with sterile PBS (BioShop Canada Inc.) and maintained in Dulbecco’s Modified Eagle Medium (DMEM F-12) without phenol red (ThermoFisher Scientific, Inc., Waltham, MA, USA) supplemented with 2% FBS (Sigma-Aldrich, Poznań, Poland).

For ER visualization the CellLight™ ER-RFP BacMam 2.0 (C10591; Invitrogen ™, Thermo Fisher Scientific, Inc., Waltham, MA, USA) was used. Cell infection with the ER-RFP BacMam 2.0 was performed two days before microscopic observation, following the manufacturer’s protocol.

### Live-cell imaging microscopy

Cell imaging was conducted using a Leica SP5 II SMD confocal microscope (Leica Microsystems, Mannheim, Germany) equipped with a Leica HCX Plan Apo 63 × lens (1.4 numerical aperture). The fluorescence signal was acquired in sequential line scanning mode using an argon ion laser (488 nm) for Citrine excitation and a laser diode (594 nm) for cell membrane and ER markers. The line average was three, and the scanning speed was 400 Hz. The fluorescence emission range was 495–570 nm for Citrine or 610–720 nm for Deep Red Fluorescence Cytopainter and CellLight™ ER-RFP. All imaging was performed on living cells in an air–steam cube incubator at 37 °C in DMEM F-12 without phenol red supplemented with 2% FBS.

### Colocalization analysis

The Coloc2 plugin in ImageJ software was used for colocalization analysis. The entire cell displaying signals from both fluorophores was selected as the ROI in all images. Only images that met the Nyquist criterion were included in the analysis, with a voxel size of xy below 93 nm for Citrine and 110 nm for RFP/Deep Red fluorophore. Background subtraction was performed using the rolling ball method with a radius of 50 pixels. The point-spread function (PSF) was determined using the equation PSF = d/ “pixel spacing,” where d = λ/2NA. The “pixel spacing” represents the distance between the centers of two adjacent pixels, and NA refers to the objective numerical aperture. The Pearson Correlation coefficient was calculated using the default threshold for background correction. A total of 3–4 independent experiments were conducted, and images were collected for analysis.

### FLIM-FRET measurements

Fluorescent lifetime imaging microscopy was conducted on cells transiently transfected with Gαi_3_ Citrine (donor) or Gαi_3_ Citrine and D_2_R mCherry (donor–acceptor) constructs. The FLIM acquisition was performed using a confocal laser scanning microscope (Leica SP5 II SMD, Germany) equipped with the PicoHarp 300 Time-Correlated Single Photon Counting (TCSPC) module (PicoQuant, Berlin, Germany). A pulsed laser diode at 470 nm (Leica, 40 MHz, Germany) was used to excite the fluorescence of the energy donor (Citrine). The emission was collected in the range of 500–550 nm using a bandpass filter, and an avalanche photodiode was employed for detection. The acquisition time for each measurement was 3–4 min, and the images were recorded in a 512 × 512 format.

Before measuring the Citrine fluorescence intensity decay, the fluorescence intensity levels of the fluorophores in cells expressing either the donor alone or the donor–acceptor pair were confirmed (*n* = 5). The analysis focused on the fluorescence signal originating exclusively from the plasma membrane, and a double exponential decay function was used for analysis in the SymPhoTime software (PicoQuant, Berlin, Germany). The reduction in fluorescence lifetime, observed as a result of FRET, was primarily evident in the short lifetime component (*τ*_1_), while the other component (*τ*_2_) remained relatively stable. This indicates that energy transfer occurred only from one donor species characterized by the lifetime *τ*_1_. Therefore, only the *τ*_1_ component was considered for calculating the FRET efficiency.

The FRET efficiency was determined using the equation: E = 1 − (*τ*_DA_/*τ*_D_), where *τ*_DA_ represents the lifetime of the donor in the presence of the acceptor, and *τ*_D_ represents the lifetime of the donor without the FRET acceptor.

### cAMP levels measurements

The intracellular cAMP levels were determined using the cAMP ELISA chemiluminescent kit (STA-500, Cell Biolabs Inc, San Diego, CA, USA), following previously described methods [20]. In brief, cells transfected with vectors encoding Gαi_3_, Gβγ dimer combinations, and D_2_R were stimulated for 10 min in a medium containing 1 μM sumanirole maleate and phosphodiesterase inhibitors (Sigma-Aldrich, Poznań, Poland). Nontransfected cells that were stimulated served as control conditions for HEK293 cells, aiming to minimize the impact of endogenous G proteins and GPCRs. The concentration of cAMP was normalized to control values in each experiment. Samples were measured in duplicates (*n* = 4, or *n* = 1–2 for Gβ_2_ trimers).

### RT-qPCR

The experiments were conducted following our previously published protocol [21]. HEK293 cells were prepared in a manner analogous to microscopy preparations. Total RNA was extracted using RNA Extractol reagent (EURx). Reverse transcription was performed with RevertAid reverse transcriptase (Thermo Scientific) according to the manufacturer's instructions, utilizing oligo(dT)_16_ primers (Genomed). The resulting cDNA was then utilized in a qPCR reaction using an Eco Real-Time PCR apparatus (Illumina). The reaction mixture consisted of 10 μl, including 5 μl of Luminaris Hi Green (Thermo Scientific), 1 μl of the resulting cDNA, and 0.3 μM specific primers (Table S1, Genomed). The reaction conditions were as follows: 95 °C for 10 min, followed by 40 cycles of 95 °C for 15 s, 60 °C for 30 s, and 72 °C for 30 s. Melting curves were analyzed for each pair of primers (95 °C for 15 s, 55 °C for 15 s, and temperature increase to 95 °C by 1 °C every 3 s). Transfections, RNA extractions, and RT-qPCR experiments were performed in three independent replicates. Each qPCR reaction was performed in duplicate. The expression levels of the β and γ subunits of G proteins were determined by analyzing the threshold cycle for each set of primers in material derived from transfected HEK293 cells, comparing it to nontransfected HEK293 cells. Data were analyzed using the ΔΔCq method with a Pfaffl modification [24] to account for PCR reaction efficiency. The results were presented as fold change in gene expression normalized against the GAPDH gene and compared to endogenous expression in the nontransfected control.

### Bioinformatic analysis

The docking of the Gβ_1_γ_1_ or Gβ_1_γ_2_ dimer to Gαi_3_ or Gαs was performed using the HADDOCK 2.4 webserver [25, 26]. PDB files of Gαi_3_ (7E9H), Gαs (7F55), Gβ_1_γ_1_ (1TBG), and Gβ_1_γ_2_ (5UZ7) were prepared according to the software developers. Residual water, ions, ligands, double occupancies of amino acid residues (keeping only the first conformation), and other amino acid chains such as receptors or other partners present in the structure were removed using PyMol. For each trimer, two scenarios were analyzed: interaction between the N-helix of the Gα subunit and amino acids N88 and N89 of the Gβ subunit, or interaction between the N-helix and residues 205–215 of the Gα subunit and amino acids N88, N89, L117, D228, D246, and W332 of the Gβ subunit [27]. The results of all investigated arrangements were visually verified by comparison with published structures, and the model with the highest score was presented. The visual representation was prepared using Discovery Studio software, version 4.0 (BIOVIA, D. S., San Diego, CA, USA, 2015).

The human Gβ and Gγ protein sequences were aligned using Clustal Omega (CLUSTAL O(1.2.4) EMBL-EBI) [28, 29]. The amino acid sequences were obtained from the UniProtKB database: Gβ: P62873, P62879, P16520, Q9HAV0, O14775; Gγ: P63211, P59768, P63215, P50150, P63218, O60262, Q9UK08, O14610, P50151, P61952, Q9UBI6, Q9P2W3. The results are presented as a Percent Identity Matrix created by Clustal 12.1.

### Statistical analysis

In all experiments, outliers were identified using Grubbs’s test and subsequently excluded from the analysis. The distribution of the data was assessed using the Shapiro–Wilk test, analysis of skewness and kurtosis, and the equality of variances was evaluated using Levene’s test. For data that followed a normal distribution, an unpaired *t*-test was conducted, and the results are presented as mean ± SEM. In cases where the data did not exhibit a normal distribution or when there were unequal sample sizes between groups, the Mann–Whitney U test was performed, and the values are reported as median ± MAD. The statistical analysis was performed using the Statistica program (data analysis software system), version 13 (TIBCO Software Inc., Palo Alto, CA, USA, 2017; http://statistica.io).

## Results

### The composition of the Gβγ dimer subunits influences the proportion of Gαi_3_ heterotrimers reaching the plasma membrane

We conducted experiments to characterize the preferences of Gαi_3_ protein for different Gβγ dimers formed by Gβ_1_ or Gβ_2_ and representative Gγ subunits from each of the five different families [6]. These include Gγ_1_, Gγ_9_, and Gγ_11_ from class I, Gγ_2_ and Gγ_8_ from class II, Gγ_7_ and Gγ_12_ from class III, Gγ_5_ and Gγ_10_ from class IV, and Gγ_13_ from class V. We tested all possible combinations of Gαi_3_ with the mentioned Gβ and Gγ subunits, except for Gαi_3_β_2_γ_1_ and Gαi_3_β_1_γ_2_ heterotrimers. It has been postulated that the Gβγ dimers in the first combination do not form a functional complex [30–32]. On the other hand, the Gαi_3_β_1_γ_2_ heterotrimer, which includes the extensively studied canonical Gβγ dimer, has already been characterized in terms of subcellular localization and functional role [20].

Previous studies have reported that all the combinations of Gβ and Gγ subunits tested in our study can form stable dimers in HEK293 cells [4]. We optimized the transfection conditions to ensure similar expression levels of all components of the heterotrimer, including Gαi_3_, Gβ, and Gγ subunits. Expression analysis using quantitative PCR for fluorescently unlabeled Gβ and Gγ subunits showed that the expression levels of Gβ_1_ and Gβ_2_ subunits in all tested combinations containing the receptor, Gαi_3_, Gβ_1_, or Gβ_2_, and the various Gγ subunits, were similar, with the relative quantity of mRNA oscillating around 1 (Fig. S1 in the supplementary information). The expression levels of Gγ subunits varied more due to the presence of different amounts of endogenous proteins in HEK293 cells. Our results are consistent with the data presented by Atwood et al. [33]. The relative amount of mRNA obtained in our experiment was higher when the endogenous mRNA of Gγ subunits was present in lower quantities in HEK293 cells. For almost all Gγ subunits, the relative mRNA amount exceeded that of Gβ subunits, since this cell line contained more endogenous mRNA of Gβ_1_ and Gβ_2_ subunits than Gγ. However, an exception was observed for Gγ_10_, whose mRNA was found to be present in the highest amount among endogenous mRNA in HEK293 cells. In this case, the result we obtained was less than zero, indicating that the expression levels we are working with are comparable to the endogenous levels.

**Fig. 1:**
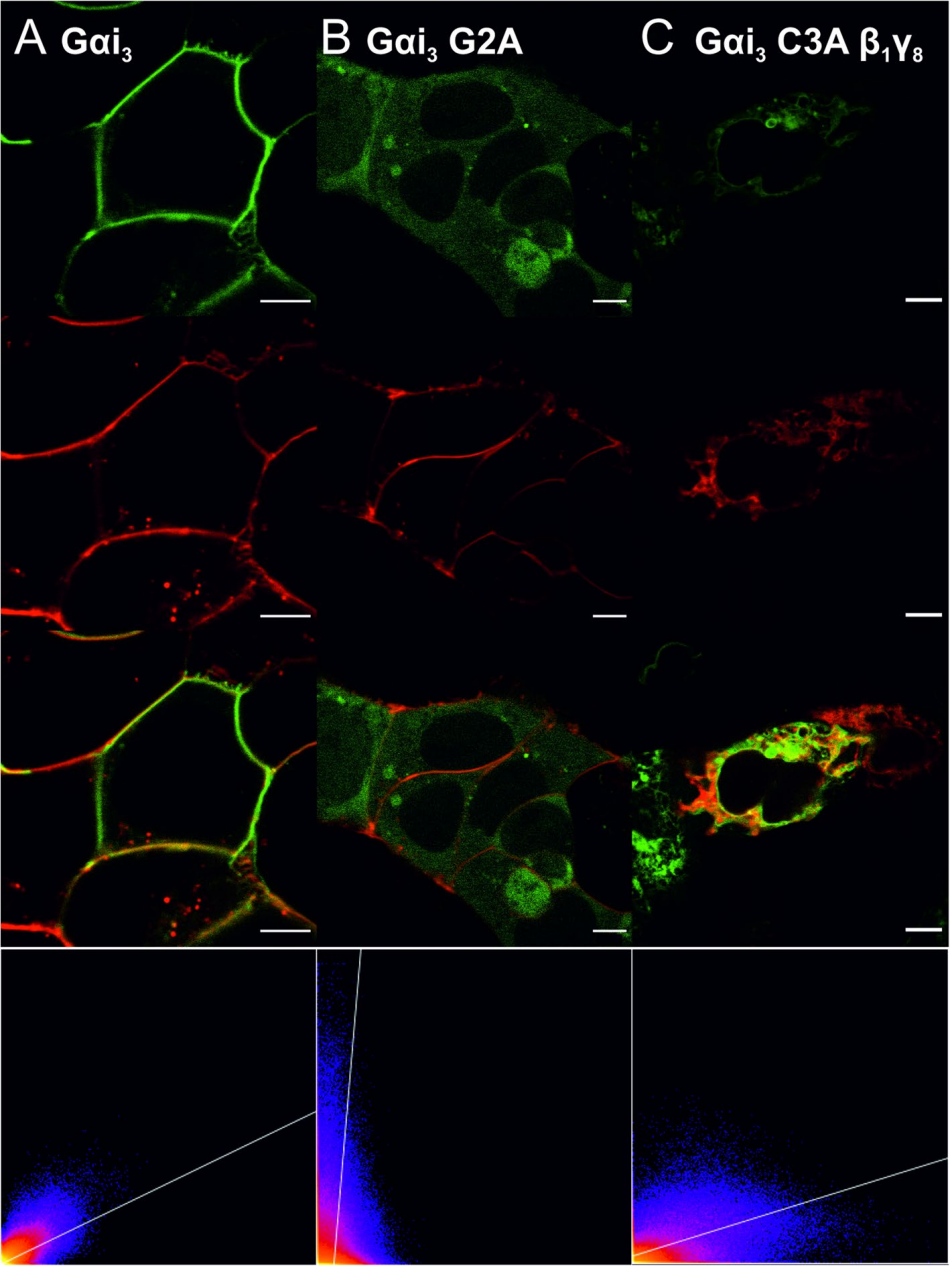
Cells transiently transfected with Gαi_3_-Citrine variants show different intracellular localization. Cells were stained with Cytopainter membrane Deep Red fluorophore (**A**, **B**) or CellLight™ ER-RFP (**C**). The scatter plot at the lower panel facilitated PCC estimation with linear regression, where the y-axis shows pixels in the Deep Red channel and the x-axis is the Citrine channel. The scale bar corresponds to 5 μm

**Fig. 2:**
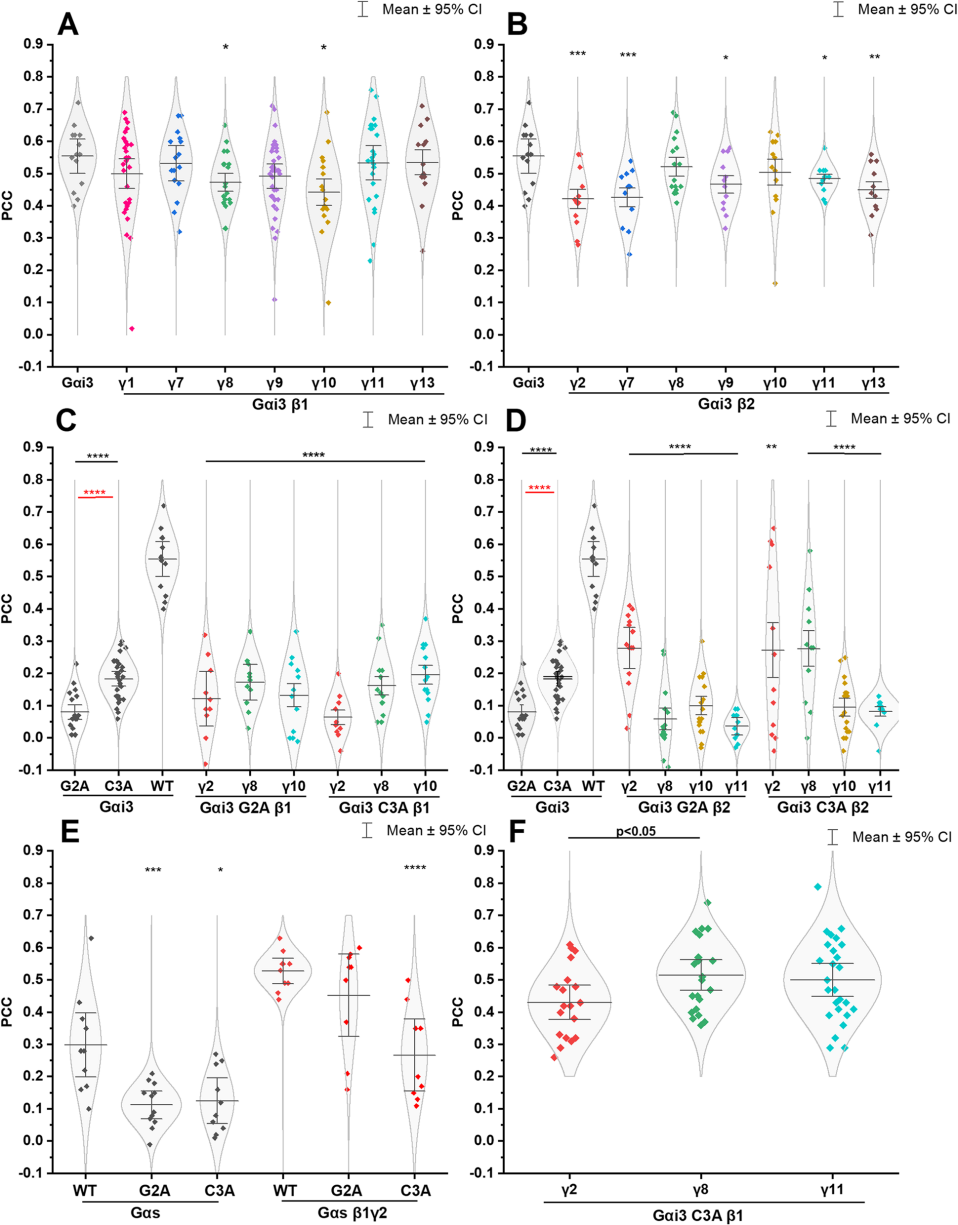
Colocalization analysis of the wild type Gαi_3_, Gαs and their muteins with various Gβγ dimers with the cell membrane or ER represented as Pearson correlation coefficient (PCC). **A**-**E** HEK293 cells transfected with Gαi_3_/Gαs-Citrine encoding vector alone or together with different Gβγ vectors without fluorescent protein were imaged after cell membrane staining (Deep Red dye). Images were collected on living cells subsequently for Citrine and Deep Red dye fluorescence. Cells transfected only with Gαi_3_/Gαs-Citrine vector represent the membrane localization of the Gαi_3_/Gαs without overexpression of the Gβγ dimer. **F** Similarly, colocalization of the Gαi_3_ C3A-Citrine with ER was performed. ER was stained using the CellLight™ system, and images were acquired two days after infection. Data were collected from at least two independent experiments (*n* = 2) and are presented as mean ± 95% confidence interval (CI). All obtained mean PCC were compared with the WT Gαi_3_(A-D)/Gαs (**E**) PCC with unpaired *t*-test (black color, * – *p* < 0.05, ** – *p* < 0.01, *** – *p* < 0.005, **** – *p* < 0.001).Additionally differences in the PCC values between Gαi_3_ muteins G2A vs C3A (red color ****—*p* < 0.001) or Gαi_3_ C3A β_1_γ_2_ vs Gαi_3_β_1_γ_8_ (black color) all with unpaired *t*-test (**F**)

Following the expression level examination of the Gαi_3_-based heterotrimers, we proceeded to investigate their subcellular localization. In some cases, we also assessed the inhibitory potential of intracellular cAMP concentration upon D_2_R activation. To begin our studies, we examined the ability of all the investigated Gαi_3_βγ heterotrimers to target the plasma membrane. We evaluated the colocalization of Citrine-tagged Gαi_3_ with the plasma membrane marker, Deep Red. The use of Deep Red fluorophore, which exhibits a significant redshift, reduced the likelihood of signal overlap between the two fluorophores. We confirmed this by imaging HEK293 cells labeled only with Deep Red, where no signal was detected in the wavelength range specific to Citrine fluorescence. Representative images for the investigated combinations are presented in Figs. 1A-B and S2. We also analyzed the colocalization between the Gαi_3_ overexpressed alone and the plasma membrane marker as a reference for the Gαi_3_βγ combinations. The colocalization results between Deep Red and the Citrine-labeled Gαi_3_βγ are shown in Fig. 2A-B, presenting only the Pearson’s correlation coefficient values due to their insensitivity to variations in pixel intensity and offset settings between images [34].

Live-cell imaging of the investigated combinations of Gαi_3_ with Gβ and Gγ confirmed that all heterotrimers reached the plasma membrane. However, we observed that the fraction of proteins reaching the plasma membrane depended on the subunit composition (Fig. S2). Interestingly, the degree of colocalization between Deep Red and Gαi_3_ in the heterotrimeric form was not significantly different from that observed for Gαi_3_ overexpressed alone. Specifically, for the complexes containing Gβ_2_, the membrane-bound fraction was slightly lower than for Gβ_1_-containing heterotrimers and Gαi_3_ only. This was particularly evident for Gαi_3_β_2_γ_2_ and Gαi_3_β_2_γ_7_ (*p* < 0.005). The estimated PCC values for Gβ_1_ heterotrimers were comparable to those of the Gαi_3_ when overexpressed alone, indicating similar levels of membrane localization. However, it is worth noting that this pattern was not observed for Gγ_8_ and Gγ_10_, which belong to class II and IV of Gγ, respectively (Figs. 2 and S2). In fact, their heterotrimers with Gβ_1_ showed slightly poorer membrane localization (*p* < 0.05) compared to other Gαi_3_β_1_γ combinations. Conversely, the Gβ_2_ complexes with these Gγ subunits exhibited colocalization with Deep Red on the plasma membrane at the same level as the Gαi_3_ overexpressed alone. A portion of the Gαi_3_ protein is probably complexed with endogenous Gβγ proteins. However, looking at the fold change in expression levels of Gβ and Gγ subunits (Fig. S1), as well as fold change in Gαi_3_ mRNA [21], we can surmise that this constitutes only a small fraction. Nevertheless, we do not underestimate their potential contribution, and we compare the results obtained after transfecting cells with all components of the heterocomplex to the results obtained after transfection with only the plasmid containing the Gαi_3_ coding sequence. Overall, these results suggest that all Gαi_3_β_2_γ complexes, except those composed of Gγ_8_ and Gγ_10_, bind to the plasma membrane with slightly lower efficiency compared to Gαi_3_β_1_γ’s.

### Effect of various Gβγ complexes on cellular localization of Gαi_3_βγ heterotrimers

To explore the effects of the Gβγ dimer on the membrane localization of Gαi_3_, we generated modified versions of Citrine-labeled Gαi_3_ by introducing specific mutations to disrupt its lipidation. Two point mutations were introduced to replace critical amino acid residues involved in lipid attachment with alanine (G2A or C3A), resulting in the elimination of lipid moieties. Previous research has indicated that myristoylation is a crucial step for the palmitoylation of the Gαi/o subunit [35, 36]. Specifically, the G2A mutation has been shown to block the attachment of a myristic moiety by NMT transferases to the N-terminal glycine of Gα, when myristoylation is eliminated, both myristoylation and palmitoylation are expected to be removed [36–38]. However, it's worth noting that myristoylation may not always be an absolute requirement for palmitoylation, as demonstrated in certain cases. The presence of the Gβγ dimer or the presence of polybasic motifs at the N-terminus of Gα can effectively support the association of Gα subunits with the cell membrane, even when myristoylation is absent, and in some cases, it can even restore palmitoylation [35, 39, 40].

The Gαs subunit was employed as a reference protein, as extensive studies have examined the roles of lipidation and the polybasic region in its cellular membrane localization [41, 42]. The corresponding lipidation-disrupting mutations were introduced in the Gαs subunit labelled with Citrine. As depicted in Fig. 2C and D and Figs. S3–S5, all four mutant proteins (Gαi_3_ C3A, Gαi_3_ G2A, Gαs C3A, and Gαs G2A) exhibited significantly weakened plasma membrane localization, as indicated by PCC values ranging from 0.08 to 0.2. This effect was particularly prominent in the case of Gαi_3_, where the wild-type protein exhibited a mean PCC value of 0.55, whereas both mutants displayed significantly reduced plasma membrane localization.

Interestingly, a notable difference (*p* < 7.3E − 6) was observed between the mutant lacking myristoylation (Gαi_3_ G2A) and those lacking palmitoylation (Gαi_3_ C3A). The mutant lacking myristoylation exhibited a lower proportion of proteins reaching the plasma membrane compared to the mutants lacking palmitoylation alone. This finding supports the widely accepted hypothesis that myristoylation alone is insufficient for attaching Gαi/o proteins to the membrane. Although this study cannot confirm the absence of palmitoylation in the Gαi_3_ G2A mutant, the poorer membrane localization observed suggests the potential absence of both lipid anchors. However, previous studies by Degtyarev et al. have demonstrated the presence of Gαi_1_ G2A in the membrane fraction and the incorporation of [^3^H]palmitate when Gβ_1_γ_2_ is present [39]. Therefore, the small portion of Gαi_3_ G2A mutant observed in the plasma membrane may be attributed to the formation of heterotrimers with endogenous Gβγ dimers in HEK293 cells and subsequent palmitoylation.

In the case of Gαs (Figs. 2E and S5), even the wild-type protein exhibited weaker plasma membrane localization compared to Gαi_3_, with a PCC of 0.3. Nonetheless, impaired membrane docking was observed for both Gαs mutants (Gαs C3A and Gαs G2A), with *p* < 0.05 for Gαs C3A and *p* < 0.005 for Gαs G2A.

In summary, removing lipidations from Gαi_3_ cause more significant changes in this protein population residing at the plasma membrane, as compared to Gαs. Gαi_3_ exhibits stronger membrane localization than Gαs, as evidenced by the difference in PCC values between the wild-type proteins. Cotransfection of Gαi_3_ muteins with selected Gβ_1_γ or Gβ_2_γ dimers resulted in variations in membrane docking. Nevertheless, as shown in Figs. 2C, D, S3, and S4 none of the studied heterotrimers showed a significant improvement in the membrane-bound fraction compared to the Gαi_3_ G2A and Gαi_3_ C3A subunits when expressed alone, with comparable PCC values. The heterotrimers Gαi_3_ C3A with Gβ_2_γ_2_ and Gβ_2_γ_8_, as well as myristoylation-deficient Gαi_3_ G2A with Gβ_2_γ_2_, displayed slightly higher membrane-bound fractions, but the differences were not substantial. In contrast, in the case of Gαs heterotrimers, the PCC values substantially increased from 0.3 for Gαs alone to 0.5 for Gαsβ_1_γ_2_. Similarly, the muteins of Gαs with impaired lipidation, especially the N-terminal palmitoylation-deficient Gαs G2A, showed restored membrane localization when cotransfected with Gβ_1_γ_2_, as indicated by a PCC of 0.43.

The myristoylation-deficient Gαi_3_ G2A mutant, when overexpressed alone or in combination with various Gβγ dimers, showed a predominant random localization in the cytosol (Fig. S3). In contrast, the palmitoylation-deficient Gαi_3_ C3A mutant exhibited specific subcellular localization, particularly in the ER, when cotransfected with certain Gβ_1_-containing dimers (Fig. S4). Figures 1C and 2F depict the perinuclear localization of Citrine-labelled Gαi_3_ C3A with Gβ_1_γ_2_, Gβ_1_γ_8_, or Gβ_1_γ_11_, which colocalized with the ER marker CellLight™ ER-RFP. Live cell imaging confirmed this ER localization and allowed for the calculation of PCC values, which indicated a high colocalization between Gαi_3_ C3A and the ER with the aforementioned Gβ_1_γ dimers (PCC 0.42–0.5). Notably, this ER localization was not observed when Gβ_2_-containing heterotrimers were coexpressed with Gαi_3_ C3A (Fig. S3A).

### Effect of different Gβγ dimers on intracellular cAMP levels induced by dopamine D_2_ receptor activation

**Fig. 3:**
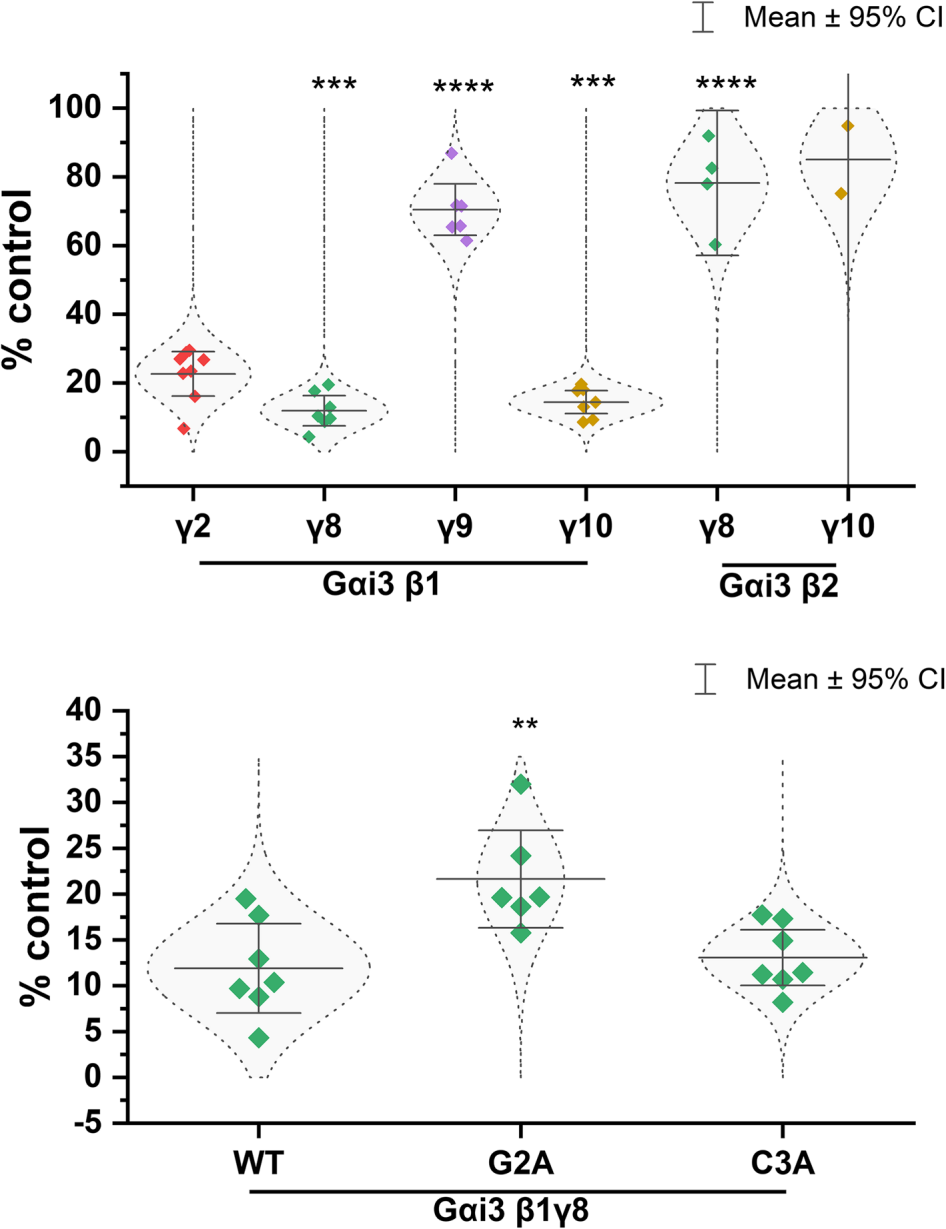
Intracellular cAMP level in the HEK293 cells lysates overexpressing Gαi_3_ with different Gβγ and for Gαi_3_ muteins after stimulation of the D_2_ receptor with full agonist (sumanirole). HEK293 cells transiently transfected with plasmids encoding different variants of the Gαi_3_-Citrine, selected Gβ and Gγ subunits and D_2_R-mCherry receptor were stimulated with sumanirole. The intracellular cAMP levels were determined in the cell lysates. Results are presented as a percentage of cAMP levels in control (stimulated nontransfected cells), as a mean ± 95% confidence interval (CI). Experiments were performed in duplicates *n* = 4, except for Gαi_3_β_2_γ_8_ (*n* = 2) and Gαi_3_β_2_γ_10_ (*n* = 1). An unpaired *t*-test was performed to evaluate differences between Gαi_3_β_1_γ_2_ vs other Gβγ dimers as well as between wild type of Gαi_3_ and muteins without lipidations in the presence of the Gβ_1_γ_8_ dimer (** – *p* < 0.01, *** – *p* < 0.005, **** – *p* < 0.001)

To assess the functional insights, we evaluated the ability of selected Gαi_3_βγ heterotrimers to transmit GPCR signals at the plasma membrane by evaluating their inhibitory influence on cyclic AMP production in response to stimulation of the dopamine D_2_R by sumanirole. We specifically focused on four Gαi_3_β_1_-containing heterotrimers with Gγ_2_, Gγ_8_, Gγ_9_, or Gγ_10_, as well as two heterotrimers containing Gαi_3_β_2_ with Gγ_8_ or Gγ_10_. We noticed noteworthy differences not only between complexes that differed in the Gβ subunit but also those that differed solely in the Gγ subunit. Interestingly, cells expressing Gαi_3_β_2_γ_8_ or Gαi_3_β_2_γ_10_ heterotrimers did not show significantly greater activity compared to control cells expressing only endogenous proteins, indicating that the interaction of these heterotrimers with the D_2_R can be neglected. As shown in Fig. 3A, Gβ_1_γ_8,_ and Gβ_1_γ_10_ heterotrimers exhibited even more efficient reduction of intracellular cAMP levels compared to the canonical Gβ_1_γ_2_ dimer (*p* < 0.001). In contrast, Gαi_3_β_1_γ_9_ displayed the least effective ACs inhibition among the examined Gβ_1_-containing heterotrimers. The inhibition of ACs achieved by Gαi_3_β_1_γ_8_, Gαi_3_β_1_γ_10_, Gαi_3_β_1_γ_2_, and Gαi_3_β_1_γ_9_ was 11.9, 14.4, 20, and 70.4% of the control, respectively. Notably, complexes containing Gγ_8_ and Gγ_10_ with the β_1_ subunit showed significantly higher inhibitory responses compared to the corresponding complexes containing the β_2_ subunit, despite having slightly lower membrane localization. These findings suggest that the dopamine D_2_R exhibits varying preferences for heterotrimers containing the same Gα subunit but different Gβγ dimers.

Furthermore, for the set of Gβγ subunits most effectively inhibiting cAMP accumulation, that is Gβ_1_γ_8_, we investigated the effect of mutations that prevent lipidation of Gαi_3_. We found that the Gαi_3_ G2A mutant (Fig. 3), which lacks myristoylation, exhibited a weaker response in inhibiting ACs compared to the wild-type Gαi_3_ (*p* < 0.01) and the Gαi_3_ C3A mutant. Interestingly, the palmitoylation-deficient Gαi_3_ C3A mutant showed equal effectiveness in reducing intracellular cAMP levels compared to the wild-type protein, and no impairment was observed. This finding is surprising considering that the membrane localization of heterotrimers containing the lipid-deficient Gαi_3_ mutants was significantly lower than that of the wild-type protein. Additionally, a substantial portion of the palmitoylation-deficient Gαi_3_ C3A mutant was found to be retained in the ER. Despite these limitations, the palmitoylation-deficient Gαi_3_ C3A mutant still exhibited an effective reduction of intracellular cAMP levels when complexed with Gβ_1_γ_8_ dimers.

### Different Gαi_3_βγ heterotrimers show differences in nanoscale distribution in the plasma membrane

Although colocalization analysis enables the investigation of interactions of the studied macromolecules in their cellular context, it does not provide the spatial resolution required to evaluate the precise nanoscale arrangement of molecules within the plasma membrane. Therefore, in order to further investigate and verify the differences observed in the colocalization analysis, we employed the FLIM-FRET method. This technique allowed us to study the membrane organization of selected Gαi_3_βγ heterotrimers relative to the D_2_R and determine the distances between them within the plasma membrane.

In this experimental setup, we monitored the fluorescence lifetime of the donor fluorophore (Citrine-labeled Gαi_3_) in the absence and presence of the acceptor (mCherry labeled D_2_R), as described in the Materials and methods section (FLIM-FRET measurements). HEK293 cells were cotransfected with the corresponding Gβ and Gγ subunits to reproduce a fully functional system. We previously confirmed the appropriate cellular localization and activity of the mCherry-labeled dopamine D_2_R [20, 21]. We focused on four heterotrimers: Gαi_3_β_2_γ_8_, which showed a high level of colocalization with the plasma membrane marker; Gαi_3_β_1_γ_8_ and Gαi_3_β_1_γ_9_, which showed the lowest level of colocalization; and the canonical trimer containing the Gβ_1_γ_2_ dimer. The decrease in the fluorescence lifetime of the donor fluorophore indicates the close proximity of proteins when both the energy donor and acceptor are present in the system.

**Fig. 4:**
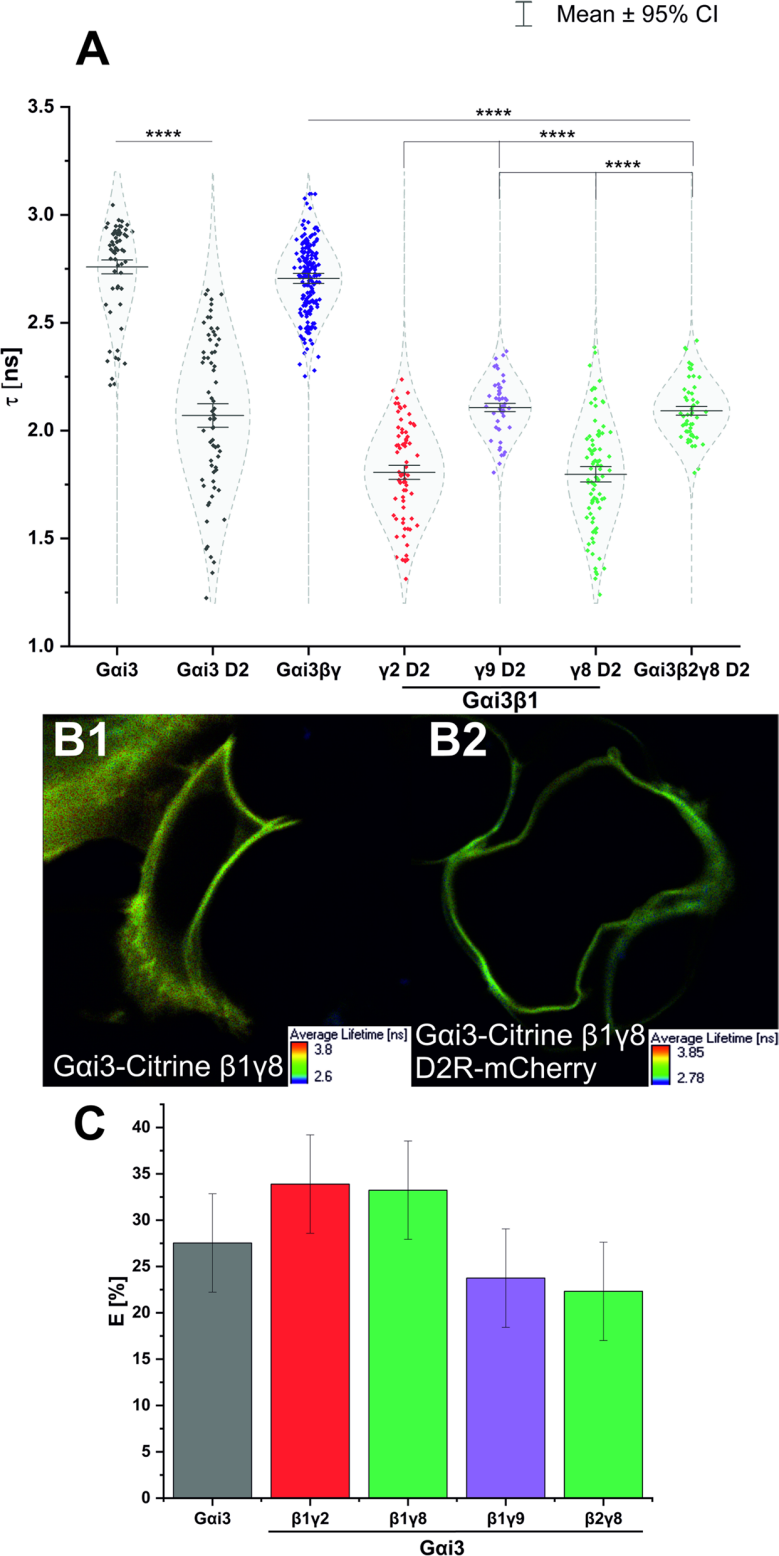
FLIM-FRET results. **A** Fluorescence lifetimes for the donor (Gαi_3_-Citrine) and the donor in the presence of the acceptor (D_2_R-mCherry). Data were collected from at least five independent experiments (*n* = 5) and are presented as mean ± 95% confidence interval (CI). The mean fluorescence lifetime of donor Gαi_3_-Citrine was compared with the donor fluorescence lifetime in the presence of the acceptor D_2_R-mCherry with an unpaired *t*-test (**** – *p* < 0.001). Because of the varying number of repeats, the median donor lifetime in the Gαi_3_βγ setup was compared to donor–acceptor systems with different Gβγ dimers using the Mann–Whitney U test (**** – *p* < 0.001). **B** Representative fluorescence lifetime images for one of the FRET donor (Gαi_3_-Citrine β_1_γ_8_; B1) and FRET donor–acceptor pair (Gαi_3_-Citrine β_1_γ_8_ – D_2_R-mCherry; B2). **C** Energy transfer efficiency (% *E*) between investigated Gαi_3_-Citrine – D_2_R–mCherry pairs; error estimated with exact differential

The FLIM images collected from cells cotransfected with Gαi_3_βγ and D_2_ showed a reduction in the apparent fluorescence lifetime of the donor (depicted as change in color toward the blue hues across all pixels), compared to those expressing only Citrine-labelled Gαi_3_βγ or Gαi_3_ (Fig. 4B). Figure 4A presents the fluorescence lifetime distributions of the fluorescence donor for all tested arrangements, including Gαi_3_ alone or in the presence of various Gβγ complexes, with or without the dopamine D_2_R. The minimal reduction in donor lifetime in FRET pairs was observed for Gαi_3_β_2_γ_8_ or Gαi_3_β_1_γ_9_ with D_2_R. Importantly, these changes were not significantly different from those observed between the Gαi_3_ was overexpressed with D_2_R only, indicating a greater distance between these heterotrimers and D_2_R compared to the other two heterotrimers and D_2_R. The calculated efficiencies of energy transfer (Fig. 4C) indicate that the spatial distribution of closely related Gαi_3_ heterotrimers differs. If the Gβ_1_γ_2_ or Gβ_1_γ_8_ dimer is present in the heterotrimer, the FRET signal is more pronounced, suggesting that Gαi_3_ is located in closer proximity to D_2_R within the membrane compared to its complex with Gβ_2_γ_8_ (where only the Gβ subunit is changed) or Gβ_1_γ_9_ dimer. Notably, these FRET results align with the measurements of intracellular cAMP levels, where the inhibition of ACs was most effective for Gαi_3_β_1_γ_2_ or Gαi_3_β_1_γ_8_. Lower FRET efficiency was observed for Gαi_3_ heterotrimers that showed less effective ACs inhibition.

One of the key findings of this study is that the nanoscale arrangement of G proteins within the plasma membrane influences the effectiveness of signal transduction. The distribution of these proteins within the membrane is more significant than the overall amount of heterotrimer bound to the membrane. Despite the Gαi_3_β_1_γ_8_ complex showing the lowest degree of colocalization with the plasma membrane marker among the Gβ_1_-containing heterotrimers, it was found to be the most effective in reducing intracellular cAMP concentration. FRET measurements revealed that, for this complex, the distance between the receptor and the Gαi_3_ subunit was the shortest, indicating a close spatial arrangement that facilitates efficient signal transduction.

### The Gβγ dimer affects the conformation of the entire heterotrimer complex

The observed differences in the interaction of heterotrimers with the plasma membrane or D_2_R can be attributed to variations in the individual subunits of the G protein complex. While the Gβ_1_ and Gβ_2_ subunits share a high similarity of 90.3% in their amino acid sequences, the significance of the Gβγ dimer becomes even more apparent when comparing the similarities between different Gγ subunits. These Gγ subunits not only differ in their attached lipid anchor but also in their amino acid sequences, particularly in the N-terminal helix region. Sequence analysis of Gγ subunits reveals a greater degree of diversity among the representatives compared to the relatively higher similarity observed in the case of Gβ subunits (Fig. S6B).

The three classes of Gγ subunits: class I (Gγ_1_, Gγ_9_, and Gγ_11_), class II (Gγ_2_, Gγ_3_, Gγ_4_, and Gγ_8_), and class III (Gγ_7_ and Gγ_12_) exhibit similarities of around 70% within their respective groups (except for Gγ_3_ in class II). However, for class IV, containing Gγ_5_ and Gγ_10_, the similarity drops to around 53%. Furthermore, the sequence of Gγ_13_ significantly differs from that of other Gγ subunits, and the similarity of class I (Gγ_1_, Gγ_9_, and Gγ_11_) to other classes is also relatively low.

**Fig. 5:**
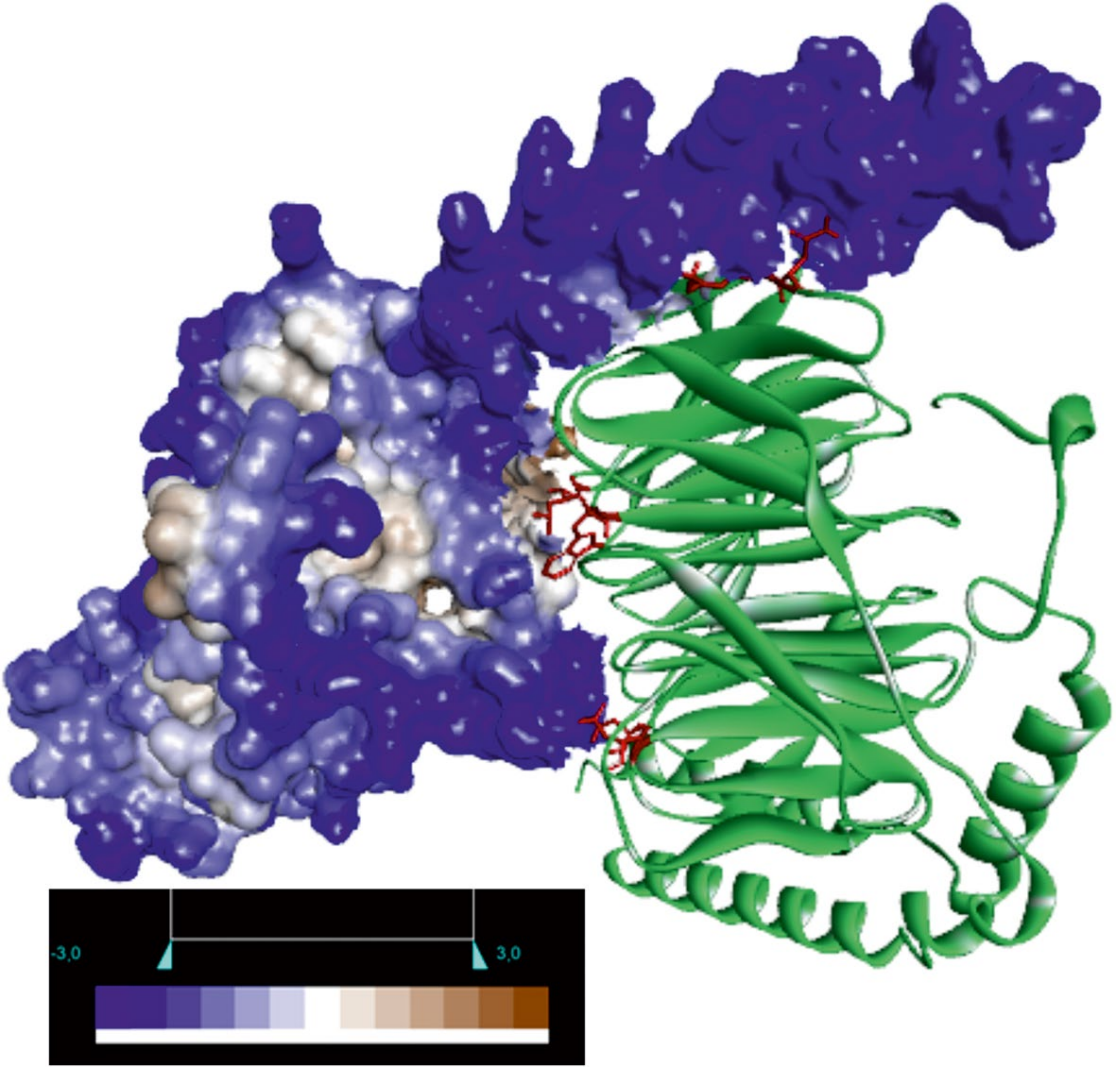
Representation of Gαsβ_1_γ_2_ docking results for interactions with all proposed Gβ_1_ binding sites. Gαs is shown as a molecular surface colored based on the hydrophobicity of the amino acid residues, the polar residues are colored blue and the hydrophobic residues are colored brown. The Gβ_1_γ_2_ dimer is colored green with selected active Gβ_1_ residues marked in red

To identify potential binding sites and determine the binding affinities of the docked poses of Gαi_3_ with Gβ_1_, we utilized HADDOCK, a molecular docking prediction server. Additionally, we performed a comparative analysis by docking the Gαs subunit. We examined four different complexes: Gαi_3_β_1_γ_2_, Gαi_3_β_1_γ_1_, Gαsβ_1_γ_2_, and Gαsβ_1_γ_1_. Our analysis focused solely on the amino acid residues within the Gβ subunit, excluding any residues within Gγ that could potentially contribute to heterotrimer formation. The selection of Gβ interface residues was based on mutational analysis data of Gβ_1_ [27]. Specifically, residues N88 and K89 were chosen due to their proximity to the Gα N-helix, while residues L117, D228, D246, and W332 were selected for their proximity to the helical fragment within the Gα helical domain (Fig. 5). We conducted two docking procedures, targeting either the N-helix of Gα that participates in the interaction with Gβ residues N88 and K89, or the entire sequence of Gα along with Gβ residues N88, K89, L117, D228, D246, and W332. Interestingly, the docking analysis of Gαs and Gαi_3_ complexes with Gβ_1_γ_2_ and Gβ_1_γ_1_ revealed slight differences in the orientation of Gβ_1_ between each complex. The best scoring pose for each complex is shown in Fig. S6A.

Table 1 presents HADDOCK scores indicating a stronger interaction between Gαs and Gβ_1_γ_2_ compared to Gαs and Gβ_1_γ_1_ (179.5 ± 9.8 for Gβ_1_γ_2_ vs 122.8 ± 9.3 for Gβ_1_γ_1_). Additionally, other important docking evaluation parameters such as cluster size, RMSD, and Z score also favor Gβ_1_γ_2_ (Table S2). While the docking analysis of both full-length Gα and the N-terminal helix alone suggests the significance of both Gα interfaces in forming a complex with Gβ_1_, our findings suggest that the N-helix of Gαsβ_1_γ_2_ plays a more prominent role in the interaction. Interestingly, this is not observed in the case of the Gαsβ_1_γ_1_ complex, where both surfaces are similarly involved in the interaction. However, for the Gαsβ_1_γ_2_ heterotrimer, the interaction between the Gα subunit and the Gβγ dimer is the strongest (HADDOCK score 179.5 ± 9.8) and preferred over the Gβ_1_γ_1_ dimer (HADDOCK score 122.8 ± 9.3). Furthermore, our results indicate that the Gαsβ_1_γ_2_ heterotrimer is slightly more stable and preferable than Gαi_3_β_1_γ_2_ (HADDOCK score 141.5 ± 10.2). However, the analysis of the docking in the case of Gαi_3_β_1_γ_2_ or Gαi_3_β_1_γ_1_ (HADDOCK score 138.2 ± 8.2) shows negligible preferences between these two dimers for Gαi_3_. This may be due to weaker preferences of Gαi_3_ towards a specific Gβγ dimer or the possibility that the Gβγ dimers used in the docking analysis may not be the most suitable choice. The analysis presented in this study highlights the potential of computational tools like AlphaFold to predict new Gβγ dimer structures for use in docking studies. These results suggest that despite considering only the amino acid residues of Gβ, the results for the Gβ_1_γ_1_ and Gβ_1_γ_2_ dimers differ. Overall, further investigation is required to fully comprehend the preferences of Gαi_3_ towards different Gβγ dimers and the potential implications of these preferences on G protein signaling.

**Table 1:** Docking results of trimer formation for Gαs and Gαi_3_ with Gβ_1_γ_2_ or Gβ_1_γ_1_ dimers

			HADDOCK score
Gαs	N	Gβ_1_γ_2_	-153.1 ± 8.0
	all		-179.5 ± 9.8
	N	Gβ_1_γ_1_	-75.3 ± 21.7
	all		-122.8 ± 9.3
Gαi_3_	N	Gβ_1_γ_2_	-61.3 ± 13.0
	all		-141.5 ± 10.2
	N	Gβ_1_γ_1_	-75.8 ± 10.1
	all		-138.2 ± 8.2

## Discussion

The precise mechanisms underlying the signaling diversity of GPCRs are not yet fully understood. One important contributing factor is the ability of GPCRs to interact with different types of G proteins, which can activate distinct downstream signaling pathways. Additionally, the existence of multiple subtypes of G protein subunits further enhances signaling diversity. However, whether GPCRs exhibit a preference for specific Gβ and Gγ subunits has not been extensively investigated.

The specificity of GPCRs for G proteins is primarily determined by the Gα subunit, but multiple isoforms of both Gβ and Gγ subunits add to the diversity of signaling. The question of whether GPCRs demonstrate a preference for particular Gβ and Gγ subunits remains unanswered. Emerging evidence suggests that GPCRs exhibit unique specificity for G proteins, not only favoring specific Gα subunits but also specific Gβγ dimers [4, 43, 44]. As previously reported, heterotrimers composed of Gαi_3_β_1_γ_9_ exhibited less efficient modulation of basal cAMP levels in HEK293 cells upon D_2_R activation compared to Gαi_3_β_1_γ_2_ [20]. The diversity of heterotrimer compositions is vast due to the abundance of proteins in each subunit group, allowing for numerous Gαβγ combinations in theory. However, not all heterotrimers are physiologically relevant, likely due to variations in expression levels or tissue localization, and the reduced stability and proven dissociation of some Gα and Gβγ combinations over extended periods of time [45, 46]. Despite recent discoveries, data on many G protein heterotrimers is still lacking. Recent studies have demonstrated that Gβ and Gγ exhibit certain preferences in forming Gβγ complexes, and the composition of the Gβγ dimer influences the kinetics and efficacy of GPCR responses [4]. As expected, each Gα subunit also demonstrates a preference for different Gβγ dimers.

In this study, we present, to our knowledge, the first comprehensive analysis of the cellular localization of Gαi_3_-based heterotrimers formed by either Gβ_1_ or Gβ_2_ in combination with representative Gγ subunits, as well as their interactions with the dopamine D_2_R. Initially, we conducted a direct comparison of the subcellular localization of Gαi_3_-based heterotrimers using the two most predominant Gβ types in cells, namely Gβ_1_ and Gβ_2_, along with several Gγ subunits from different classes. This approach was chosen regardless of downstream signaling, as it can be challenging to reliably compare all heterotrimers based solely on downstream signals.

To assess the subcellular localization of the studied heterotrimers, we utilize live cell fluorescence imaging and colocalization strategies. In order to generate heterotrimers with a specific subunit composition, we utilized the strategy of overexpressing the complex components in HEK293 cells. It is important to note that we ensured the level of overproduction was not excessively high but sufficient to preferentially produce heterotrimers with the desired composition. G proteins are typically transported to the plasma membrane as full heterotrimeric complexes, which assemble on the cytoplasmic surface of the Golgi apparatus or ER, and it is widely accepted that heterotrimer assembly occurs before the acylation of the Gα subunit [47, 48]. Previous studies have indicated that all combinations of the analyzed Gβ and Gγ subunits interact with other members of the Gαi/o family, particularly the Gαi_1_ subunit [45]. Furthermore, Gβ subunits demonstrate distinct preferences for Gγ subunits. Additionally, certain combinations of Gβγ dimers have been observed to be relatively less stable and tend to dissociate into their individual Gβ and Gγ components when isolated from the cellular environment [4, 45, 49, 50].

Our data reveals that all examined Gαi_3_ heterotrimers primarily localize at the plasma membrane, with minimal fluorescence signal detected from within the cell. However, we observed a slightly lower fraction of Gβ_2_-containing complexes bound to the plasma membrane compared to Gβ_1_, despite their highly similar structure (with a sequence identity of 90%) and generally interchangeable molecular interactions [51]. Previous studies have demonstrated that Gβ_1_ can interact equally well with most Gγ subunits in the presence of exogenous Gαo_A_. On the other hand, Gβ_2_ exhibits greater selectivity and significant differences in binding to specific Gγ subunits, including Gγ_1_, Gγ_7_, Gγ_8_, and Gγ_11–13_ [4]. Another study utilizing the yeast two-hybrid assay showed that the examined Gγ subunits (Gγ_1–5_ and Gγ_7_) could interact with both Gβ_1_ and Gβ_2_, albeit the interaction with Gβ_2_ was relatively weaker [52].

The reduced plasma membrane localization of heterotrimers composed of Gβ_2_ observed in our experiments could potentially be attributed to differences in their interaction capabilities, with a few exceptions. For instance, the Gαi_3_β_2_γ_8_ and Gαi_3_β_2_γ_10_ heterotrimers exhibited greater efficiency in binding to the plasma membrane, even when compared to their complexes with Gβ_1_. Conversely, combinations such as Gαi_3_β_2_γ_2_, Gαi_3_β_2_γ_7_, and Gαi_3_β_2_γ_13_ showed the lowest membrane localization. Interestingly, these Gγ subunits belong to different classes and are all geranylgeranylated at the C-terminus. Recent demonstrations have shown that the Gαo_A_ subunit exhibits the strongest binding affinity for Gβ_2_γ_7_ and Gβ_2_γ_8_ while exhibiting weaker binding to Gβ_2_γ_1_, Gβ_2_γ_11_, and Gβ_2_γ_13_ [4]. Previous studies have reported that Gβ_5_γ dimers can distinguish between different defined Gα subunits [4, 53–55]. For example, they interact more preferentially with the Gαi_1_ subunit and exhibit weaker binding to Gαs [55]. Conversely, the Gβ_1_γ_2_ and Gβ_1_γ_9_ dimers preferentially bind to Gαi_3_ over Gαs [20].

The interactions between Gα and Gβγ primarily occur through two interface regions [56, 57]. The first interface is formed between the top of the Gβ propeller and Switch I and Switch II of Gα, while the second interface is located between blade 1 of the Gβ propeller and the N-terminus helix of Gα. Crystal structures of G-protein heterotrimers and Gβγ alone indicate that Gβγ subunits do not undergo significant conformational changes upon heterotrimer formation, but the available molecular data is somewhat limited, as crystal structures of G proteins have only included combinations of Gβ_1_γ_1_ or Gβ_1_γ_2_ dimers. The contact interface between Gγ and Gβ subunits is located in regions composed of residues that are generally highly conserved in both proteins [58].

Our analysis of docking heterotrimers composed of Gαs or Gαi_3_ with Gβ_1_γ_2_ or Gβ_1_γ_1_ revealed that the orientation of Gβ_1_ in heterotrimers can differ. Additionally, HADDOCK docking parameters suggest differences in the binding affinity of Gα subunits to Gβγ dimers. While Gα subunits exhibit a high degree of sequence and structural conservation, there are minor differences in the Gβγ contact interface regions between them. Gαi_3_ and Gαs differ primarily in their N-terminus helix sequence, with some differences also present within the conserved Switch I and Switch II regions. Consequently, these amino acid residue differences within the interface regions of both Gα and Gβ subunits may potentially impact their interaction. Unlike Gβγ, Gα undergoes structural changes upon heterotrimer formation. The binding of Gβγ induces a rearrangement of Switch II and induces conformational changes within Switch I and Switch II [59]. Furthermore, in the heterotrimeric state, the N-terminal helix of Gα remains intact, as it is stabilized by interactions with Gβ [56, 60].

The G protein complex is targeted to the plasma membrane through multiple binding signals that act synergistically to achieve high affinity and specificity for interaction with membrane lipids. These signals involve a combination of covalent lipid modifications on both the Gγ and Gα subunits, as well as the presence of positively charged residues in the C-terminus (preCaaX region) of Gγ and the N-terminus of Gα [5, 21, 61]. The prenylation of Gγ subunits, along with the polybasic motif, plays a crucial role in directing to the membrane localization and dissociation of the Gβγ dimer as well as facilitating the interaction with Gα and downstream effectors [60, 62, 63]. Similarly, the myristoylation of Gα enhances its affinity to Gβγ [64, 65]. Previous studies have demonstrated that Gα is necessary for the plasma membrane localization of Gβγ. When different combinations of Gβ and Gγ are overexpressed without Gα, the Gβγ dimers exhibit weak localization at the plasma membrane and tend to accumulate in intracellular structures, predominantly in the ER [66–68].

The observation that even Gαi_3_ protein overexpressed alone localizes to the plasma membrane with similar efficiency as its heterotrimers (Figs. 2 and S2) supports the notion that Gαi_3_ plays a crucial role in directing the complete heterotrimeric complex to the membrane. This is further supported by the finding that heterotrimers containing the Gαi_3_ G2A mutein, which is resistant to lipid modification, are predominantly retained in the cytosol unless coexpressed with Gβ_2_γ_2_, which partially restores membrane localization. This suggests that the myristoylation of Gαi_3_ may not be as critical for the association of this specific heterotrimer as it is for others. It is still possible that palmitoylation of the Gαi_3_ G2A mutant occurs after complex formation, as previously observed in the case of Gαi_1_ G2A Gβ_1_γ_2_ [39]. However, the results obtained with the palmitoylation-deficient Gαi_3_ C3A mutant, which displayed a similar level of membrane association, lack conclusive evidence due to the significant dispersion of the PCC value. Nevertheless, it can be reasonably concluded that the localization of this heterotrimer to the plasma membrane relies on protein–protein interactions. Among the tested combinations of Gβγ dimers, only two Gβ_2_γ dimers, including Gγ_2_ and Gγ_8_ from the same class, were effective at rescuing the membrane localization of the Gαi_3_ C3A mutein. Importantly, none of the tested dimers containing Gβ_1_ were able to restore the membrane localization of the heterotrimer with the palmitoylation-deficient Gαi_3_. In summary, these findings suggest that palmitoylation is essential for the stable binding of Gαi_3_ heterotrimers to the plasma membrane, as previously proposed [38, 69, 70]. However, the significance of protein–protein interactions should not be overlooked. Overall, Gαi_3_ appears to be the driving force behind the effective targeting of Gαi_3_ heterotrimers to the plasma membrane, resulting from the combined effects of fatty acid modification and protein–protein interactions.

The inhibition of Gαi_3_ palmitoylation leads to the accumulation of a significant fraction of Gαi_3_ C3A in the ER when certain Gβ_1_γ combinations are present. This observation is intriguing because overexpressed Gαi_3_ C3A mutein alone typically exhibits random localization in the cytosol. These findings align with previous research showing that mutations abolishing palmitoylation cause Gα subunits to shift to the cytosolic fraction, as observed in the cases of Gαo, Gαz, and Gαi_1_ [38, 71, 72]. Another study demonstrated that when palmitoylation of Gαi_2_ is prevented, either through mutation of the palmitoylated cysteine residue to serine or treatment with 2-bromopalmitate (2-BP), the Gβ_1_γ_2_ dimer accumulates in the ER while the heterotrimer remains in the Golgi [67].

The trafficking pathway for G proteins involves the preassembly of heterotrimers before palmitoylation of the Gα subunit, followed by delivery of the complex to the plasma membrane [73]. A conserved family of enzymes known as DHHC-Cysteine Rich Domain (DHHC-CRD) enzymes has been identified as at least one of the enzymes responsible for the S-palmitoylation of Gα proteins. Most DHHCs localize at the Golgi, with some distributed among the ER, plasma membrane, and endosomes [74]. Previous studies have shown that DHHC3 and 7 enzymes acylate Gαq, Gαs, Gαi_2_ and Gαo subunits mainly in the Golgi [37, 75]. However, other studies have shown that DHHC3 and 7 knockout, as well as DHHC7 overexpression, had only a minor effect on Gαi_1-3_ acylation [76]. Instead, it was suggested that the acylation of these subunits may be mediated by different acyltransferases, likely localized outside the Golgi apparatus. Our experiments revealed that Gβ_1_γ_2_, Gβ_1_γ_8_, and Gβ_1_γ_11_ were capable of retaining palmitoylation-deficient Gαi_3_ C3A in the ER, indicating the involvement of ER-localized DHHC acyltransferases in the palmitoylation of the Gαi_3_ subunit. These findings suggest that multiple enzymes redundantly participate in the acylation of the Gαi family. Although S-palmitoylation is generally considered nonspecific and proximity-based, recent studies have shown that DHHCs can differentiate between substrates even with minor differences in amino acid composition [37]. The cellular distribution of palmitoylation-deficient Gαi_3_ C3A depends on the specific Gβγ dimer with which it is coexpressed. This led to the consideration that the particular Gβγ dimer incorporated into the heterotrimer may influence the positioning of the N-terminus of Gα relative to the membrane, thereby affecting its susceptibility to specific acyltransferases.

In contrast to Gαi_3_, Gαs exhibit weaker plasma membrane localization when expressed alone. The presence of Gβγ is crucial for targeting Gαs to the plasma membrane. Although only one Gβγ dimer composition, Gβ_1_γ_2_, was examined, this observation aligns with previous findings indicating that the membrane affinity of the Gαs subunit is determined by the specific Gβγ dimer [68]. Studies by Evanko and colleagues demonstrated that Gβ_2–4_γ_2–3_ dimers were effective in promoting more pronounced plasma membrane localization of Gαs. Based on the available evidence, it appears that Gαs do not play a major role in anchoring heterotrimers to the membrane, unlike Gαi_3_. Instead, there may be reciprocal cooperation between Gαs and Gβγ that facilitates the targeting of complex components in the plasma membrane.

Mutation of the N-terminal glycine or adjacent cysteine in Gαs resulted in a significant reduction in membrane localization compared to wild-type Gαs, which was partially restored by coexpression with Gβ_1_γ_2_. However, this effect was more pronounced for the N-terminal palmitoylation-deficient Gαs G2A mutein than for the second palmitoylation-deficient Gαs C3A mutant. It has been proposed that myristoylation and a polybasic motif act as complementary signals for initial membrane targeting in Gα subunits [40]. The polybasic motif, composed of positively charged amino acids on the protein surface, is more prominent in Gαs than in myristoylated members of the Gαi family (reviewed in [15]). Such positively charged regions on a protein’s surface act as membrane-binding signals through electrostatic interactions with the negatively charged headgroups of membrane lipids. Removal of positive charges from this motif leads to a shift in the localization of Gαs from the plasma membrane to the cytosol [41]. Therefore, the effectiveness of Gβ_1_γ_2_ in rescuing the membrane binding loss of the N-terminal palmitoylation-deficient Gαs G2A mutein suggests that the presence of the polybasic motif in Gαs compensates for the lack of palmitoylation. It is worth noting that in heterotrimer complexes, the importance of the second palmitoyl anchor appears to outweigh that of the N-terminal one, as evidenced by the reduced relative amount of heterotrimers containing the Gαs C3A mutein that bind to the membrane. Thus, even in the absence of N-terminal palmitoylation, the polybasic motif alone in Gαs is sufficient for the formation of a complex with Gβ_1_γ_2_ and subsequent palmitoylation at the N-terminal C3 residue. This provides direct evidence for the importance of the interaction with the Gβγ dimer and subsequent palmitoylation of Gαs at the C3 residue for the membrane targeting of Gαs heterotrimers.

In our efforts to identify Gαi_3_ heterotrimer combinations that effectively bind to the dopamine D_2_R and inhibit ACs upon activation by sumanirole, a selective full-efficacy agonist for the D_2_R [77] we discovered that certain Gβγ combinations were more successful. The most effective combination consisted of Gβ_1_ with either Gγ_8_ or Gγ_10_. Slightly less effective combinations included Gγ_2_, while the least preferred dimers were Gβ_1_γ_9_, Gβ_2_γ_8_, and Gβ_2_γ_10_. This suggests that the specific association of Gβ_1_, rather than Gβ_2_, with Gγ_8_ or Gγ_10_ enables a favorable interaction with the receptor. Therefore, the Gβ subunit plays a crucial role in presenting the Gα subunit in a suitable conformation for effective receptor interaction. Recent evidence also supports the idea that direct interaction between Gβ and the receptor acts as a scaffold, facilitating Gα subunit recruitment and localization of the G protein at the active receptor [78, 79]. Interestingly, we found no correlation between the levels of Gβγ dimers on the plasma membrane and the efficiency of ACs inhibition by the D_2_R. Instead, our FRET experiments proved that the spatial distribution of G proteins and D_2_R molecules on the plasma membrane played a more significant role. Specifically, there was a strong correlation between the involvement of specific Gαi_3_ heterotrimers and D_2_R in the cell membrane and the efficiency of ACs inhibition.

Our studies also confirmed the substantial role of the Gγ subunit in the interaction between the G protein complex and the activated receptor, consistent with previous research [6, 80–82]. Although there is limited structural data demonstrating the direct interaction of Gβγ with GPCRs [83] the crucial role of Gγ subunits in signaling is well-established. Different Gβγ complexes primarily vary in the speed and efficiency of G protein activation upon receptor stimulation. Following receptor activation, the Gβγ complex relocates from the plasma membrane to intracellular membranes [4, 82, 84]. The C-terminal helices of the examined Gγ subunits display wide sequence similarity scores ranging from 30 to 67% [5]. These subunits differ in terms of their lipidation patterns and adjacent stretches of basic and hydrophobic amino acid residues, which contribute to their ability to bind to the membrane and potentially interact with GPCRs. Among the examined subunits, only Gγ_9_ belongs to the rapidly translocating class I. Our findings for Gβ_1_γ_9_ support the hypothesis that fast-translocating Gβγ complexes are less effective in activating effectors at the plasma membrane compared to slower-moving Gβγ complexes [85].

Our findings indicate that even in the absence of post-translational palmitoylation of Gαi_3_, the Gβ_1_γ_8_-complexed heterotrimer retains its ability to effectively reduce intracellular cAMP levels, similar to the wild-type protein. However, it is noteworthy that this particular heterotrimer remains localized in the ER membrane. Interestingly, the D_2_R has been identified in the ER membrane [86]) and retains its ability to initiate G-protein signaling, even within the ER [87]. This is possible because key components of signaling pathways, including ACs, are also present in the ER and Golgi apparatus [88]. There is growing evidence suggesting that G proteins are not only present but also functional in intracellular compartments such as the ER, nucleus, endosomes, and Golgi complex [89, 90]. It is important to note that activation of GPCRs localized within these internal membranes can lead to distinct effects compared to those occurring at the cell surface [91]. In this study, we focused on assessing intracellular cAMP levels resulting from D_2_R activation and observed comparable outcomes to the stimulation of the receptor fraction located in the cell membrane. However, it is crucial to consider that overall signaling may differ between these two receptor populations, as G proteins can bind to diverse effectors to activate distinct signaling pathways. Further investigations are necessary to gain deeper insight into the specific Gβγ-effector signaling in both the subcellular localizations of the D_2_R.

## Conclusion

Our study confirmed that the composition of heterotrimers, including all subunits (Gα, Gβ, and Gγ), has a significant impact on the strength and specificity of GPCR-mediated signaling. It is crucial to recognize that an interaction between a GPCR and Gα cannot be generalized to all complexes, not only within a specific class of Gα but also among different heterotrimers of the same subunit. Each heterotrimeric complex has the potential to exhibit distinct variations in overall conformation, primarily determined by the specific combination of Gα, Gβ, and Gγ subunits. This variation has implications for the interaction between heterotrimeric complexes and GPCRs, as well as their interactions with membrane lipids.

Recent studies have postulated that selective GPCRs likely engage specific G proteins through shared contacts and further stabilize the complex by forming selective contacts located at the periphery of the GPCR:G protein interface [92]. Our findings strongly support this notion and add to the growing body of evidence emphasizing the fundamental importance of specific compositions of Gαβγ heterotrimers in GPCR signaling. Both the Gα and Gβγ subunits contribute to the modulation of downstream signaling events, highlighting their cooperative role in mediating GPCR signaling pathways.

### Supplementary Information

**Additional file 1.**

## Data Availability

All data are contained within the article and supporting information. The datasets used and analyzed data during this study are available from the corresponding author on reasonable request.
