# Supplementary material for: Beyond the G protein α subunit: investigating the functional impact of other components of the Gαi3 heterotrimers
Source: Cell Commun Signal. 2023 Oct 10;21:279. doi: 10.1186/s12964-023-01307-w (PMC10566112; doi:10.1186/s12964-023-01307-w)
Supplement: Supplementary file 2 — Additional file 1. [file 12964_2023_1307_MOESM1_ESM.pdf]

Electronic supplementary material for:

Beyond the G protein α subunit: Investigating the functional impact of other components of the $G\alpha i_3$ heterotrimers

Beata Rysiewicz, Ewa Błasiak, Paweł Mystek, Marta Dziedzicka-Wasylewska, Agnieszka Polit*

Department of Physical Biochemistry, Faculty of Biochemistry Biophysics and Biotechnology, Jagiellonian University, Gronostajowa 7, 30-387 Kraków, Poland

*Correspondence to: a.polit@uj.edu.pl; Tel.: +48 12 6646156

Table S1. List of oligonucleotides used in RT-qPCR experiments.

Protein	Forward	Reverse
GAPDH	CAACAGCGACACCCACTCCTCC	GGTCTTACTCCTTGGAGGCCATGTG
$G\beta_1$	CCTTTCTCTTGCTCCTGACACCAGAC	GTCAGCACGAAGGTCAAACAGCCT
$G\beta_2$	CACGTTTGTGTCAGGCGCC	GTAGCCGTTGGGGAAGAAAGCC
$G\gamma_1$	TTGACCAGCTCAAGAAAGAAGTGACAC	GCCAGATCGTTCTTCAACGTAATCTCTTAC
$G\gamma_2$	GCAGCTTAAGATGGAAGCCAATATCGAC	TTTTCTGAAGCCGGAACAGGGG
$G\gamma_7$	CACTAACAACATAGCCCAGGCCC	CAGCAGGGGGTCGTTCCG
$G\gamma_8$	GAAGCTGGAGGTGAACATCGACC	GGGGTTCTCCGCGGCG
$G\gamma_9$	TGGCCCAGGATCTCAGCGAG	TTGGGCCTCCACGTACTCCT
$G\gamma_{10}$	GCTCAAGTTGGAGGCTGGCG	GTTACTTCCAGCTGGAACACCCAC
$G\gamma_{11}$	ATGCCTGCCCTTCACATCGAA	AGAACATTTAGACACTTGTTGTCTCTGCAAC
$G\gamma_{12}$	CCAGCAAAACAGCAAGCACCAAC	TGGTATTCCTATCAGCAAAGGGTCACTC
$G\gamma_{13}$	AGAGGTGGAGAGCCTCAAGTACC	TTCCACCCATGGGTTGTTCTTCATC

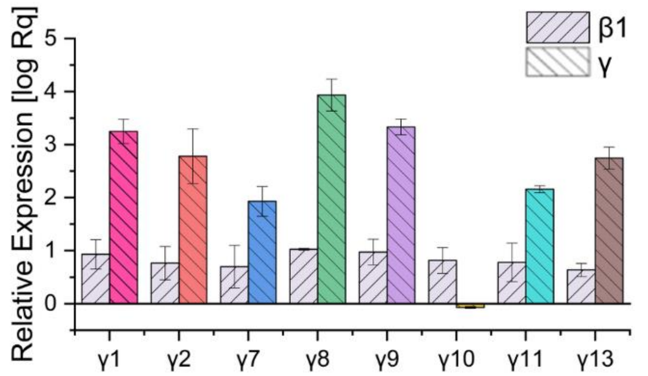

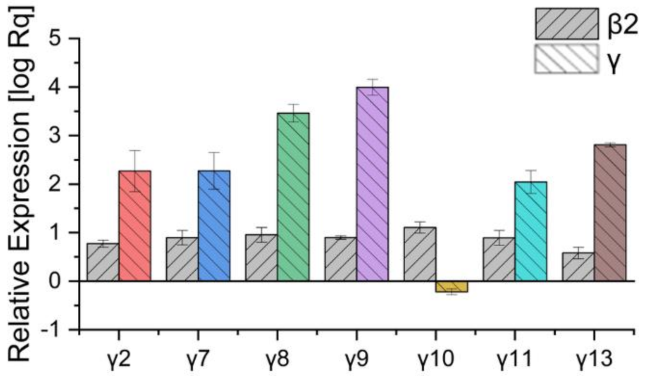

Figure S1. Relative gene expression of Gβ and Gγ subunits. Total mRNA extracted from HEK293 cells transiently transfected with Gαi_3-Citrine and D_2R-mCherry, as well as different combinations of Gβ and Gγ subunits, was used in RT-qPCR experiments. Data are presented as the logarithm of relative gene expression relative to nontransfected cells, measured in three independent experiments. Error bars are SEM, unpaired *t*-test showed no significant differences.

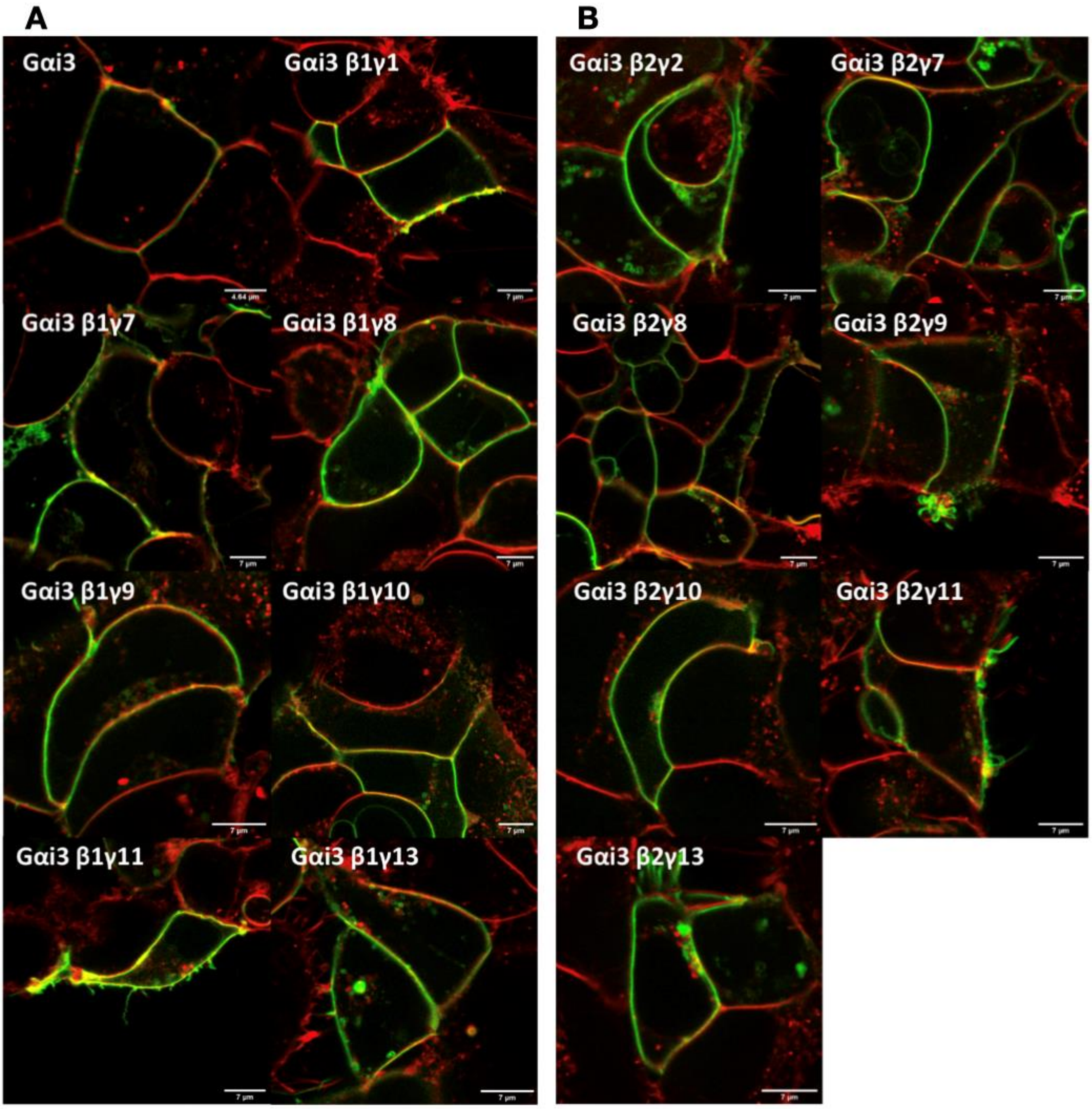

Figure S2. Representative microscopic images demonstrating the colocalization of Gαi_3β$_1$γ or Gαi_3β$_2$γ complexes with the cell membrane. Cells were stained before imaging with Cytopainter membrane Deep Red fluorophore – red, green – Gαi_3-Citrine, yellow – colour overlay. Cells were cotransfected three days before the experiment with Gαi_3 protein and (A) Gβ$_1$ subunit and various Gγ or (B) Gβ$_2$ subunit and various Gγ. The scale bar corresponds to 7 µm, except for the Gαi_3 protein itself, where the bar corresponds to 4.6 µm.

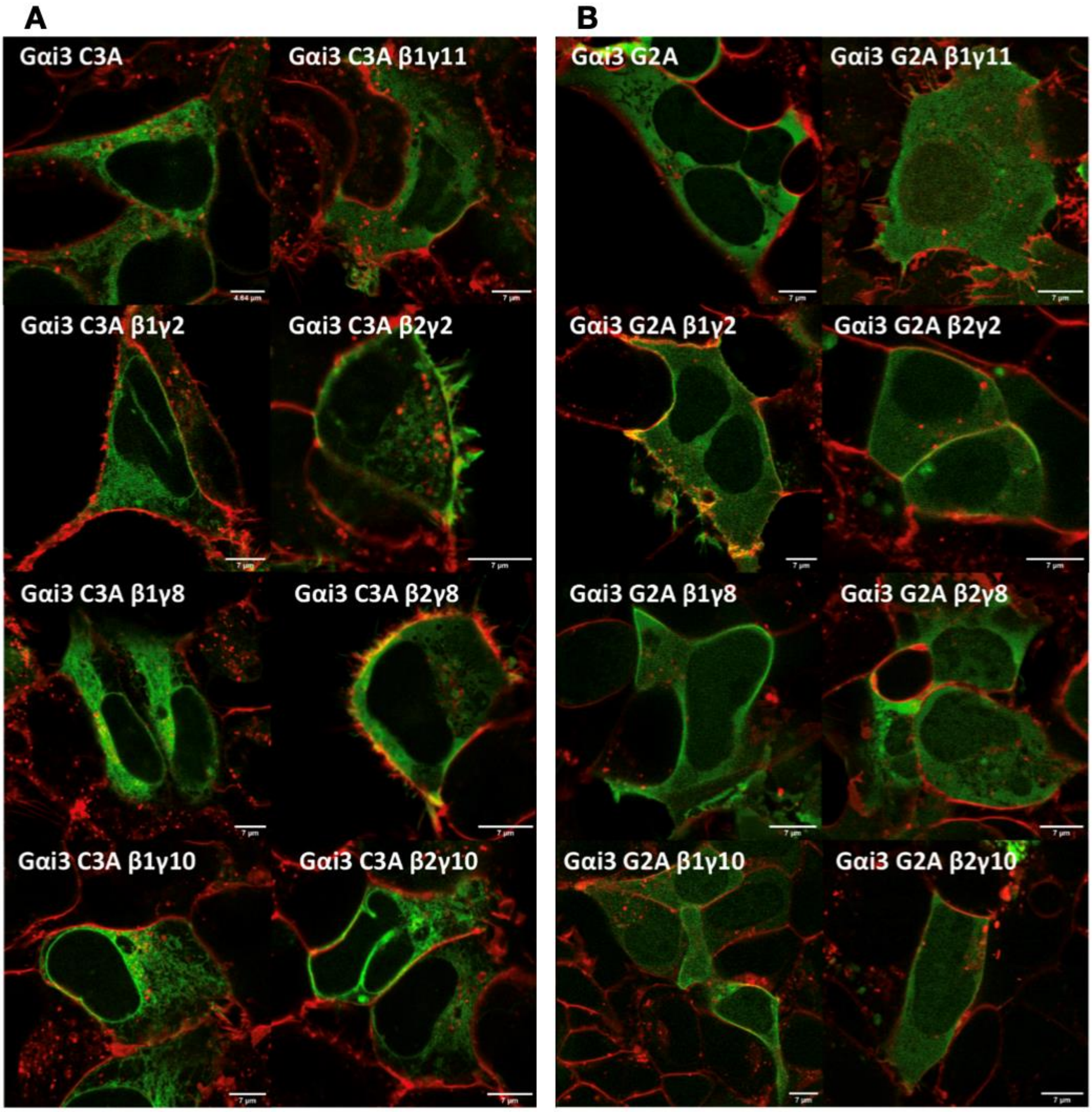

Figure S3. **Representative microscopic images demonstrating the colocalization of different heterotrimers containing Gαi_3 C3A or Gαi_3 G2A with the cell membrane.** Cells were stained before imaging with Cytopainter membrane Deep Red fluorophore – red, green – Gαi_3-Citrine variants, yellow – colour overlay. Cotransfection of Gαi_3 C3A (A) or Gαi_3 G2A (B) with different combinations of Gβγ subunits. The scale bar corresponds to 7 µm, except for the Gαi_3 C3A protein itself, where the bar corresponds to 4.6 µm.

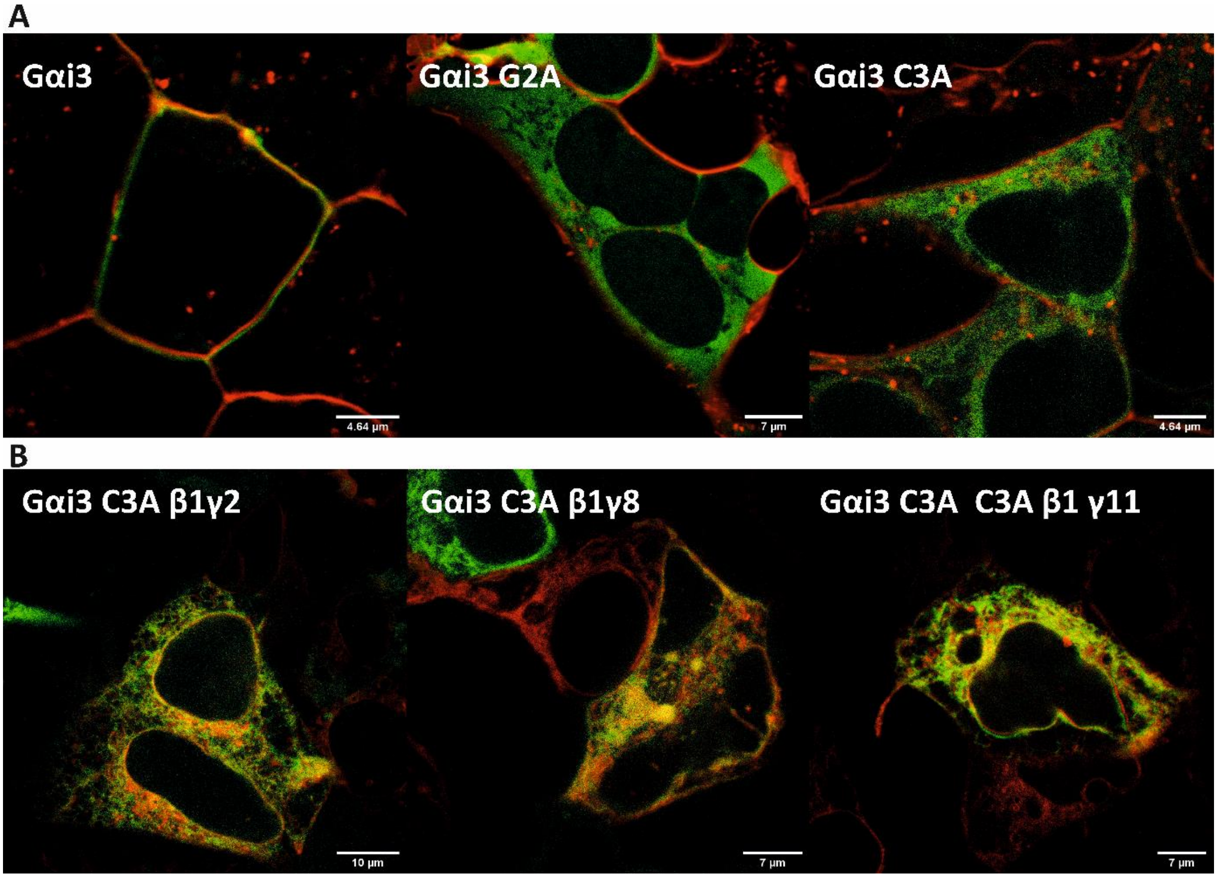

Figure S4. Representative microscopic images demonstrating the colocalization of Gαi_3, Gαi_3 G2A and Gαi_3 C3A with the cell membrane and their exemplary Gαβγ complexes with the ER. (A) green – Gαi_3-Citrine (cells three days after transient transfection), red – cell membrane stained with Deep Red (cells stained before imaging); (B) green – Gαi_3, red – ER stained with CellLight™ ER-RFP (two days after infection), yellow – colour overlay. The scale bar corresponds to different lengths: 4.6 µm for the Gαi_3 and Gαi_3, 10 µm for the Gαi_3 C3A $\beta_1\gamma_{11}$, and 7 µm for the rest proteins.

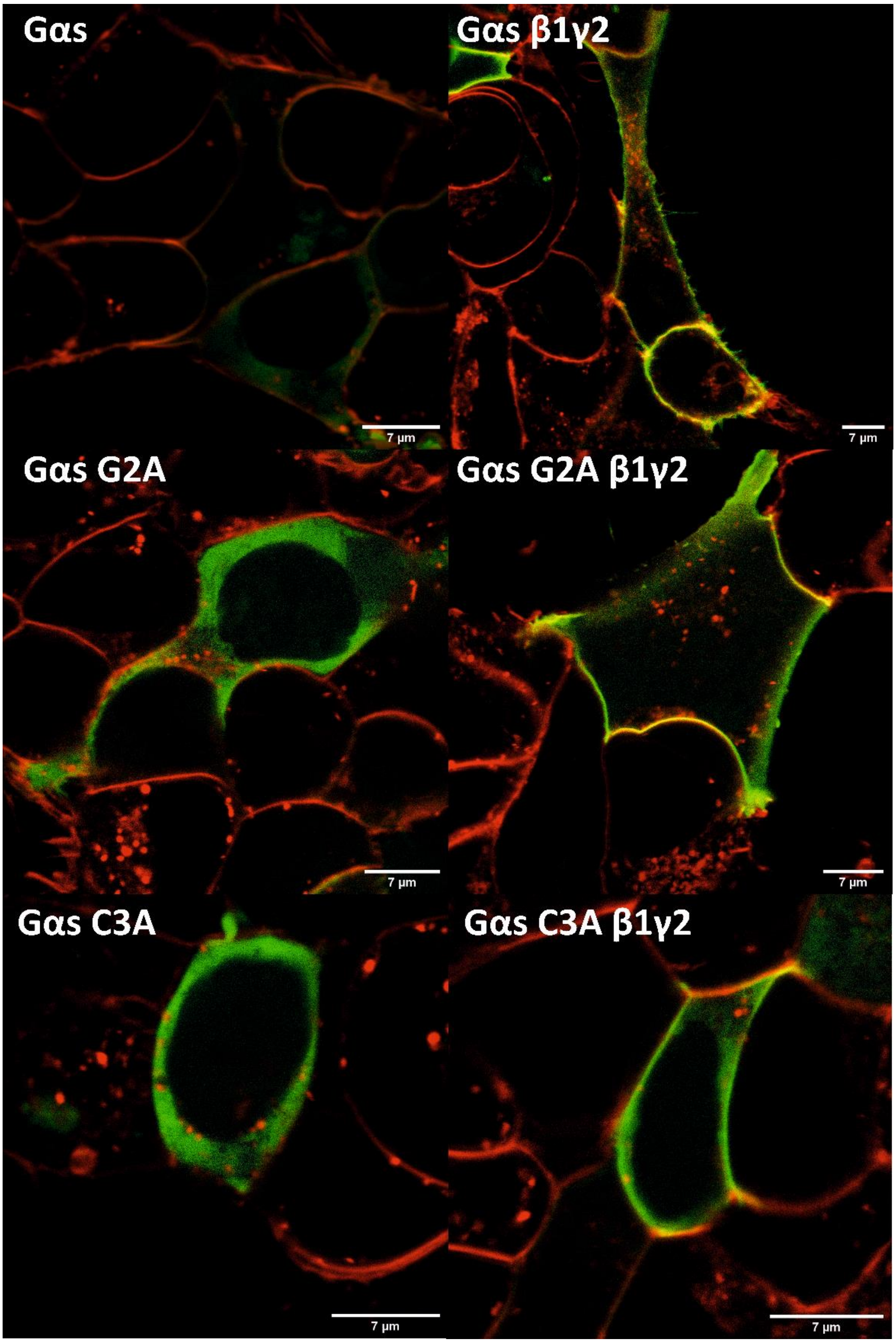

Figure S5. Representative microscopic images demonstrating the colocalization of Gαs, Gαs G2A, Gαs C3A and their complexes with G$\beta_1\gamma_2$ with the cell membrane. Cells were stained before imaging with Cytopainter membrane Deep Red fluorophore – red, green – Gαs-Citrine (cell three days after transient transfection), yellow – colour overlay. The scale bar corresponds to 7 µm, except for the Gαs protein itself, where the bar corresponds to 6 µm.

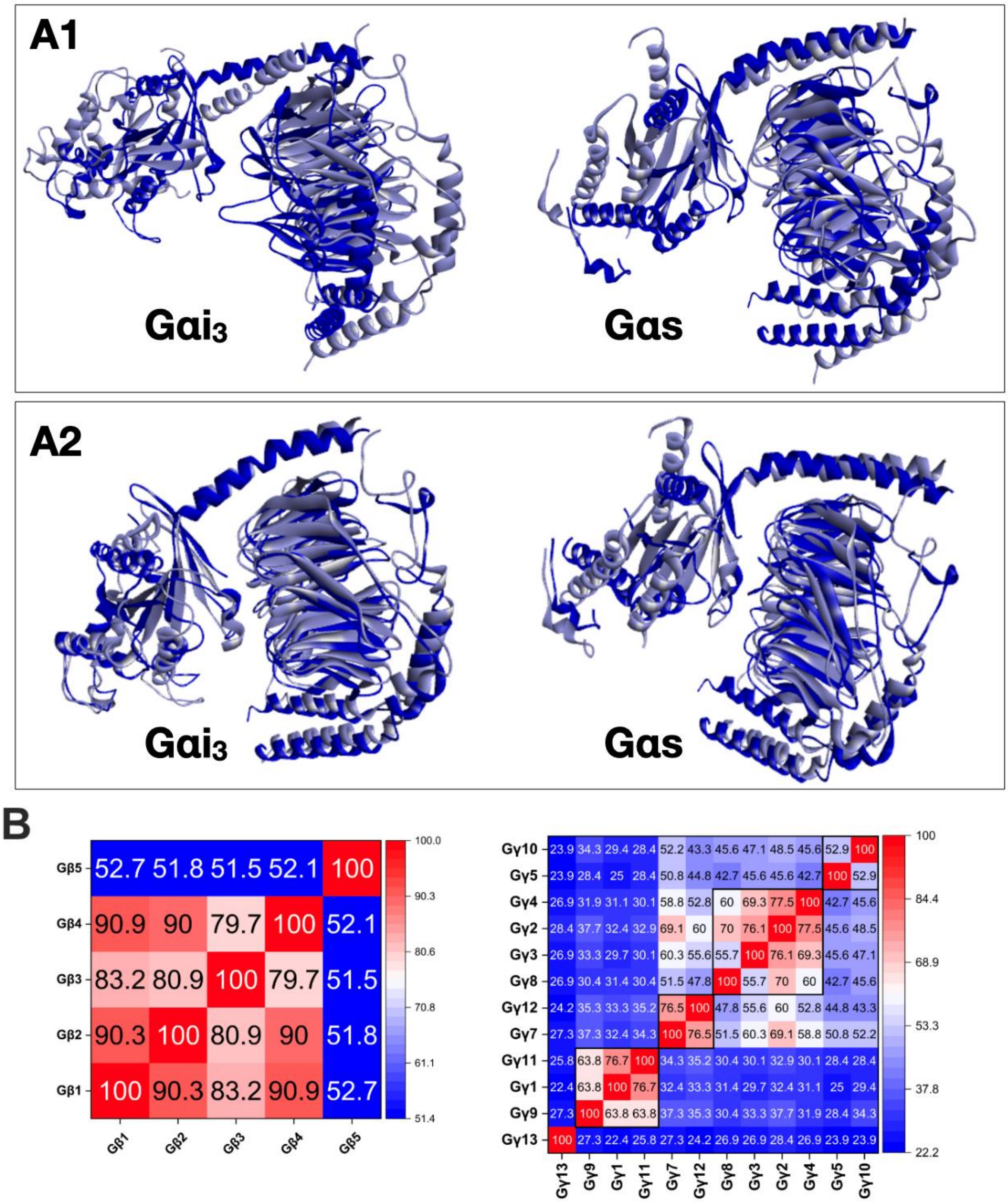

Figure S6. Bioinformatics analysis demonstrating docking of Gαi_3 and Gαs with selected areas of G$\beta_1\gamma_1$ and G$\beta_1\gamma_2$ complexes. Heat map of the human Gβ and Gγ proteins. (A) Docking was performed with HADDOCK 2.4 webserver between Gαi3 (A1, A2) or Gαs (A3, A4) and G$\beta_1\gamma$1 or G$\beta_1\gamma_2$ in two settings: considering only amino acid residues of Gβ involved in the interaction with the N-terminal helix of Gα (A1, A3) or with the addition of Gβ residues near Gα helical domain (A2, A4). The structures are presented as a superimposition of the G$\alpha\beta_1\gamma_2$ (blue) and G$\alpha\beta_1\gamma_1$ (grey) results. (B) Sequence similarity between human Gβ and Gγ subunits prepared with CLUSTAL O (1.2.4). The Gγ subunits were categorized into five classes as described in the literature.

Table S2. Docking results of trimer formation for Gαs and $G\alpha i_3$ with $G\beta_1\gamma_2$ or $G\beta_1\gamma_1$ dimers.

	$\beta_1\gamma_2$				$\beta_1\gamma_1$			
	**Gαs N**	**Gαs all**	**$G\alpha i_3$ N**	**$G\alpha i_3$ all**	**Gαs N**	**Gαs all**	**$G\alpha i_3$ N**	**$G\alpha i_3$ all**
**HADDOCK score**	**-153.1 +/- 8.0**	**-179.5 +/- 9.8**	**-61.3 +/- 13.0**	**-141.5 +/- 10.2**	**-75.3 +/- 21.7**	**-122.8 +/- 9.3**	**-75.8 +/- 10.1**	**-138.2 +/- 8.2**
Cluster size	8	18	5	30	5	11	4	40
RMSD from the overall lowest-energy structure	0.8 +/- 0.5	1.0 +/- 0.6	5.9 +/- 1.2	0.7 +/- 0.5	13.7 +/- 0.3	3.8 +/- 0.3	2.3 +/- 1.7	1.0 +/- 0.6
Van der Waals energy	-47.7 +/- 4.9	-63.6 +/- 7.6	-30.6 +/- 4.7	-55.9 +/- 7.1	-41.9 +/- 13.8	-44.6 +/- 7.0	-33.4 +/- 7.3	-38.0 +/- 5.7
Electrostatic energy	-670.7 +/- 41.3	-741.6 +/- 46.7	-250.4 +/- 45.3	-467.2 +/- 33.5	-335.2 +/- 40.4	-497.9 +/- 37.3	-411.0 +/- 49.5	-559.0 +/- 57.3
Desolvation energy	-6.9 +/- 5.9	-11.0 +/- 4.2	-2.8 +/- 1.6	-14.1 +/- 4.2	2.5 +/- 8.4	-1.8 +/- 4.1	18.5 +/- 3.8	-17.3 +/- 1.4
Restraints violation energy	356.2 +/- 24.1	434.1 +/- 26.7	221.7 +/- 43.5	218.6 +/- 50.6	311.1 +/- 83.2	231.9 +/- 50.7	212.9 +/- 76.3	289.8 +/- 67.5
Buried Surface Area	2470.0 +/- 58.4	2911.8 +/- 120.0	1522.7 +/- 60.9	2262.8 +/- 143.7	1889.0 +/- 184.7	2369.8 +/- 70.4	1944.6 +/- 211.1	2209.6 +/- 141.6
**Z-Score**	**-2.8**	**-2.7**	**-0.7**	**-1.4**	**-0.8**	**-1.5**	**-1.6**	**-2.0**
